# Around the Clock: Wearing a Flex-Printed trEEGrid Electrode Patch and Miniaturized Amplifier for Nearly 24 Hours Across Daytime and Overnight EEG Sessions

**DOI:** 10.3390/s26165309

**Published:** 2026-08-21

**Authors:** Joanna E. M. Scanlon, Axel H. Winneke, Wiebke Pätzold, Karen Insa Wolf

**Affiliations:** Fraunhofer Institute for Digital Media Technology IDMT, Branch Hearing, Speech and Audio Technology HSA, 26129 Oldenburg, Germany; axel.winneke@idmt.fraunhofer.de (A.H.W.); wiebke.paetzold@idmt.fraunhofer.de (W.P.); karen.insa.wolf@idmt.fraunhofer.de (K.I.W.)

**Keywords:** long-term monitoring, mobile EEG, electrode patch, wearable, cognitive load

## Abstract

**Highlights:**

**What are the main results?**

- The flex-printed trEEGrid patch was combined with a miniaturized, modularized amplifier prototype, which allowed for wearing the unobtrusive multichannel EEG for nearly 24 h and recording during cognitive tasks, daily life, and home-based sleep.
- The trEEGrid patch showed comparable signal quality to conventional Ag/AgCl electrodes in signal-to-noise ratio and demonstrated robust event-related potential components (P3, N1) and spectral markers (alpha, beta, theta) on both recording days and times.

**What are the main implications of the results?**

- The trEEGrid patch can be worn for up to 24 h, allowing EEG monitoring at any time of day and even sleep recording.
- This new EEG technology can help to deepen our understanding of how the brain works over long time periods, in daily life environments and at different times of the day, with comfortable unobtrusive monitoring for better ecological validity.

**Abstract:**

Long-term EEG measurements (over eight hours) can allow a deeper understanding of everyday life brain functioning. However, most EEG systems can only be used for short periods in controlled settings, due to limitations in comfort, signal quality of EEG electrode sensors and amplifier size. In this study, we measured brain activity during day and night on ten lab member participants using our new flex-printed trEEGrid patch electrodes and a miniaturized and modularized amplifier prototype. The patch was left on participants up to 24 h while they went about their day. EEG was recorded during several exploratory tasks and overnight. Daytime scenarios included auditory and visual oddball tasks, as well as a resting-state measurement and sudoku cognitive load task. Impedances were recorded throughout all tasks. Ag/AgCl ‘ring’ electrodes were used as comparison. Daytime tasks were performed twice (i.e., once in the afternoon and again the next morning), to assess signal quality changes over time. P3 oddball, resting state and workload-related spectral effects were observed on both days. Auditory N1 amplitudes were similar between the patch and ring electrodes. The system shows promising data quality over the nearly 24 h wearing time and allows new possibilities for daytime and sleep EEG studies.

## 1. Introduction

Electroencephalography (EEG) allows millisecond-level temporal resolution, enabling researchers and clinicians to link neural activity to specific stimuli. This characteristic makes EEG particularly valuable for studying brain function in ecologically valid settings beyond the traditional laboratory or clinic. For example, EEG’s high temporal resolution can be used to detect transient neurophysiological events either linked to external stimuli in the environment or internally generated events such as epileptic bursts or spikes as well as brief sleep spindles or k-complexes. Furthermore, long-term data, such as sleep EEG, can provide information about a person’s state of health. Finally, in the field of epileptology, long-term monitoring is of particular interest. To date, this has only been feasible for multi-channel EEG in a clinical setting, which places a significant burden on the patient. Translating such extended measurements to everyday environments remains largely infeasible. A key bottleneck is the lack of electrode sensors that can maintain both adequate signal quality and wearing comfort over prolonged periods of time.

Conventional EEG electrodes typically consist of Ag/AgCl sintered ring electrodes which require gel or moistened sponges to bridge the connection between the skin and the electrode. However, this gel tends to dry out over time, leading to discomfort and poor or even loss of signal. Additionally, these rigid metal electrodes and attached wires make the system inconvenient for long-term wear. The application of conventional electrodes is furthermore time-consuming and requires trained personnel, which limits their usage outside of laboratories and medical environments. Reduced channel EEG systems such as cEEGrids [1,2] use flex-printed, light-weight circuit boards (flex-PCBs) which adhere directly to the hairless skin around the ear. These lightweight systems have been shown to have significant advantages for long-term EEG recordings, with stable signal quality as well as comfort [1]. Despite the restriction of only being useable on hairless skin (e.g., around the ears, temples, forehead, etc.) flex-printed electrodes have been demonstrably effective for measuring EEG signal features including event-related potentials (ERPs) such as the P3 response [1,2,3], alpha oscillations [4], as well as sleep signals [5,6] and epileptiform activity [2]. The concept behind the flex-print electrode grid cEEGrid has been further developed regarding carrier material and design into the so-called trEEGrid [7]. This flex-printed version has been used to observe changes in EEG frequency spectra linked to vigilance changes over time during simulated driving [8].

However, these flex-printed electrode grids still require electrode gel to be applied right before application on the skin, which limits self-applicability. To progress towards a long-term self-applicable solution to record EEG, da Silva Souto et al. [7] used pre-gelled neonatal ECG electrodes which allowed for self-application and adequate sleep staging from the participants’ homes. We built upon this concept by combining the flex-printed trEEGrid with pre-applied conductive material at electrode sites instead of electrode gel, which we call a ‘trEEGrid patch’ [9]. The current study uses the patch (i.e., electrode sites equipped with pre-applied conductive material) with a different material from da Silva Souto et al. [9], in combination with a prototype of a miniaturized and modularized amplifier (Brain Products GmbH, Gilching, Germany). The current study also extends the patch’s wearing time from 5 to 6 h to nearly 24 h.

In the current study, participants wore our trEEGrid patch for nearly 24 h, performing four different exploratory tasks on two days in a row, along with a sleep recording. The tasks included visual and auditory oddball tasks, a resting state task and the well-known puzzle game of Sudoku at two different difficulty levels, to observe brain activity in different conditions. The Sudoku task was chosen to investigate different levels of cognitive load in a common everyday task. During the tasks, classical Ag/AgCl electrodes were used alongside the trEEGrid patch to compare both in terms of the morphology of the event-related potentials (ERP) and signal-to-noise ratio (SNR). For the rest of the day, no EEG or impedance was recorded but the patch was left attached. The same tasks from the afternoon on day 1 were repeated in the morning on the second day. Over-night recording at participants’ homes was completed between day 1 and day 2. Participants filled out comfort and sleep quality questionnaires to obtain subjective ratings regarding the trEEGrid patch and the mini amplifier.

Our first hypothesis was that the EEG system (trEEGrid patch plus mini amp) would show typical robust brain signals on both days, including a significant P3 amplitude difference between standards and deviants in the auditory and visual oddball tasks [10,11], as well as the typical increase in EEG alpha power during eyes-closed compared to eyes-open resting state data [12]. Our second hypothesis was that we would have high enough quality to compare brain activity during two levels of a Sudoku task and resting state, as well as a general analysis of the participants’ sleep patterns. Our third hypothesis was that participants would feel relatively comfortable wearing the system, even after 24 h.

## 2. Materials and Methods

### 2.1. Participants

Ten participants were recruited from the Mobile Neurotechnologies working group at Fraunhofer for this study (mean age = 35, age range = 27–49 years, 5 women). Lab members were chosen to participate because the amplifier was in a prototype stage and required careful handling, especially for usage during overnight recordings at their homes. For example, the 3-D printed casing and the on/off switch were still somewhat fragile. Participants had normal hearing and vision, or corrected vision, and had no history of neurological diseases or medical implants. All participants provided written consent. Protocols in this study were approved by the local ethics board at the Carl von Ossietzky University of Oldenburg (reference number Drs.EK/2025/035).

### 2.2. Materials

**Figure 1:**
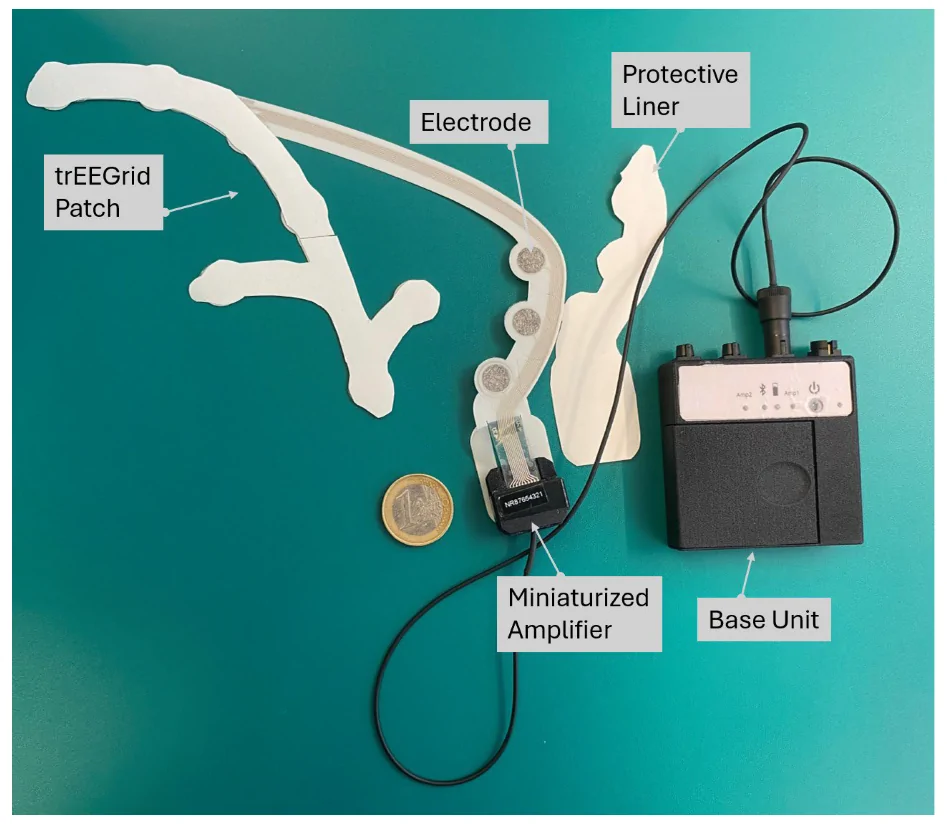
EEG system with the miniaturized amplifier attached to the trEEGrid patch, which is partly covered by a protective liner. The amplifier is linked via cable to the base unit. Euro coin for scale.

EEG recording was done using a trEEGrid patch with pre-applied conductive material (FLX070271—Flexcon^®^ Omni-Wave™ TT 200 BLACK H-510 400 POLY H-9 44PP-8, Flexcon Company, Inc., Spencer, MA, USA). The trEEGrid’s adhesive design requires hairless skin areas for the electrodes to stay in place and maintain good contact to the skin. The trEEGrid patch design includes eight recording electrodes with two additional electrodes serving as reference and ground channels. Electrodes 1, 3, and 5 can also allow the derivation of vertical, horizontal and diagonal electrooculogram (EOG) signals, in addition to EEG recordings, similar to the EOG recordings in sleep laboratories [7]. The miniaturized amplifier system (mini amp) for the patch system consists of two elements: an eight-channel mini-amplifier with direct connection to the patch placed behind the ear and a base unit with battery and Bluetooth module for connection to a recording laptop. The concept was developed in collaboration with Brain Products GmbH, who produced this initial version. Figure 1 shows the EEG system with the trEEGrid patch and the miniaturized and modularized amplifier system. The mini amplifier is directly attached to the trEEGrid patch.

**Figure 2:**
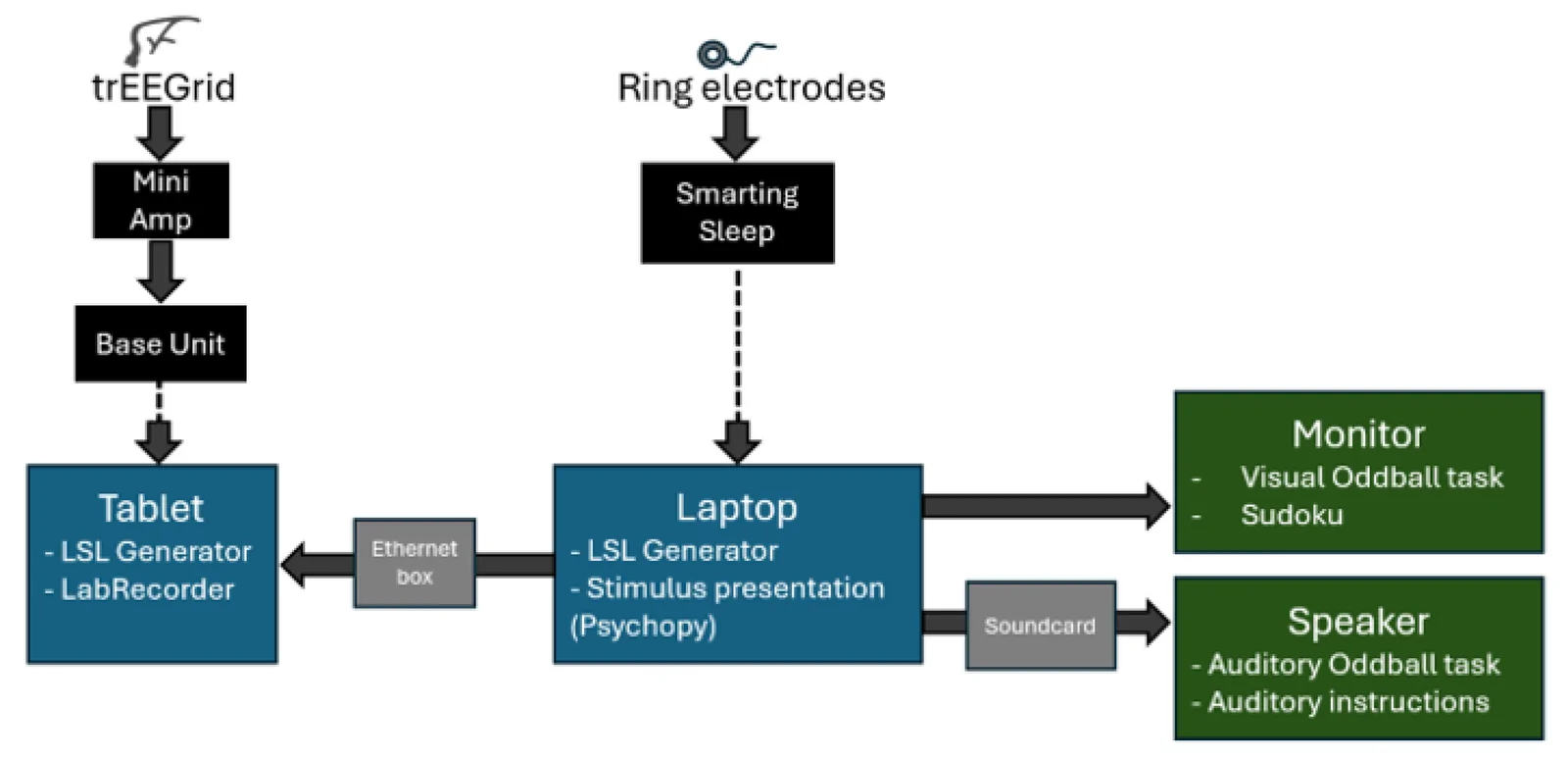
Connections between devices used in the experiment. Dotted lined arrows indicate wireless connections and block arrows indicate wired connections.

Figure 2 shows a schematic visualization of the devices used in the experiment. All data streams were temporally synchronized and collected using LabStreamingLayer (LSL; [13]). trEEGrid patch data was collected via the mini amp prototype and connected via Bluetooth to a tablet computer (Dell Latitude 7320 Detachable, Dell Technologies Inc., Round Rock, TX, USA) for the generation of the LSL stream based on a software program provided by Brain Products GmbH (BrainVision LSL generator). Ring electrode data was collected via the smarting sleep amplifier (mBrainTrain, Belgrade, Serbia), which was also connected via Bluetooth to a laptop (Dell BHC Latitude 5450, Dell Technologies Inc., Round Rock, TX, USA) for the generation of the LSL stream based on a program by mBrainTrain (Smarting App). This laptop also was used for presenting the experimental stimuli, using a monitor for visual stimuli and a soundcard (RME Fireface UC, RME Audio, Haimhausen, Germany) and speaker for auditory tasks. Both the tablet and laptop were connected via an ethernet cable and box. The recording was done with the LabRecorder [14] on the tablet.

### 2.3. Set-Up and Experimental Procedure

Participants were sent information on how to prepare for the experiment, including a consent form, before the experiment began. They were asked to get plenty of rest, to avoid using make-up or excessive skin products, and, if applicable, to wear glasses instead of contacts to reduce blinking. Participants who shave their face regularly were asked to do so on the day of the experiment, because some electrodes would be placed on areas that may have facial hair.

**Figure 3:**
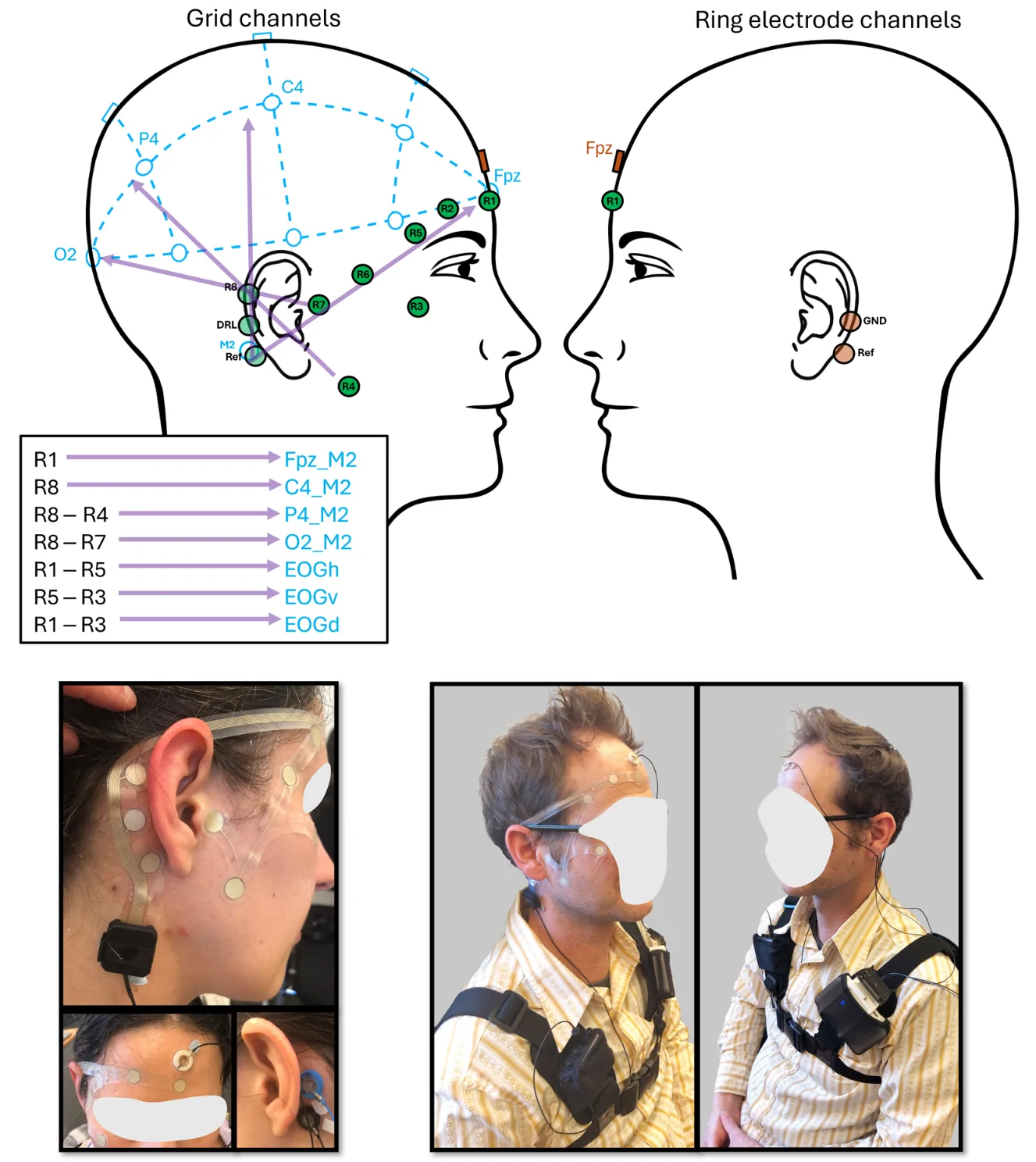
Study set-up. (**Top**): trEEGrid (green) and ring electrode (orange) channel locations, and spatial approximation equations (see Section 2.6.3). (**Bottom**): EEG system set-up including both amplifiers, battery pack and chest belt.

Upon arrival, participants were asked to review and sign the consent form. We then briefed them again about the specific contents of the study and answered any remaining questions. Prior to placing the EEG systems, participants’ skin was cleaned with abrasive electrolyte gel (Abralyt HiCl, Easycap, Wörthsee, Germany) and alcohol. The trEEGrid patches were placed on the right side of each participant’s face, as shown in Figure 3. As reference for the signal quality estimation standard Ag/AgCl ring-electrodes were placed on the forehead, at location Fpz, with the ground and reference electrodes on the mastoid behind the participants’ left ear. The signal recording with ring electrodes was done with a mobile EEG amplifier (Smarting Sleep, mBrainTrain, Belgrade, Serbia). The ring electrodes were prepared with abrasive electrolyte gel (Abralyt HiCl, Easycap, Wörthsee, Germany).

Each participant performed four EEG tasks in the afternoon of day 1 and repeated them in the morning of day 2 of the study. Participants also recorded their EEG during sleep between day 1 and 2. They kept the trEEGrid patch and amplifier on from the beginning to the end of the experiment, but removed the ring electrodes at the end of the first day and re-applied them on the second day.

### 2.4. Questionnaires

Participants filled out questionnaires on the comfort level of the trEEGrid patch and the amplifier at the end of the first day, as well as in the morning and afternoon of the second day. They also filled out a sleep quality questionnaire before bed in the evening of the first day and in the morning of the second day.

### 2.5. Experimental Tasks

**Figure 4:**
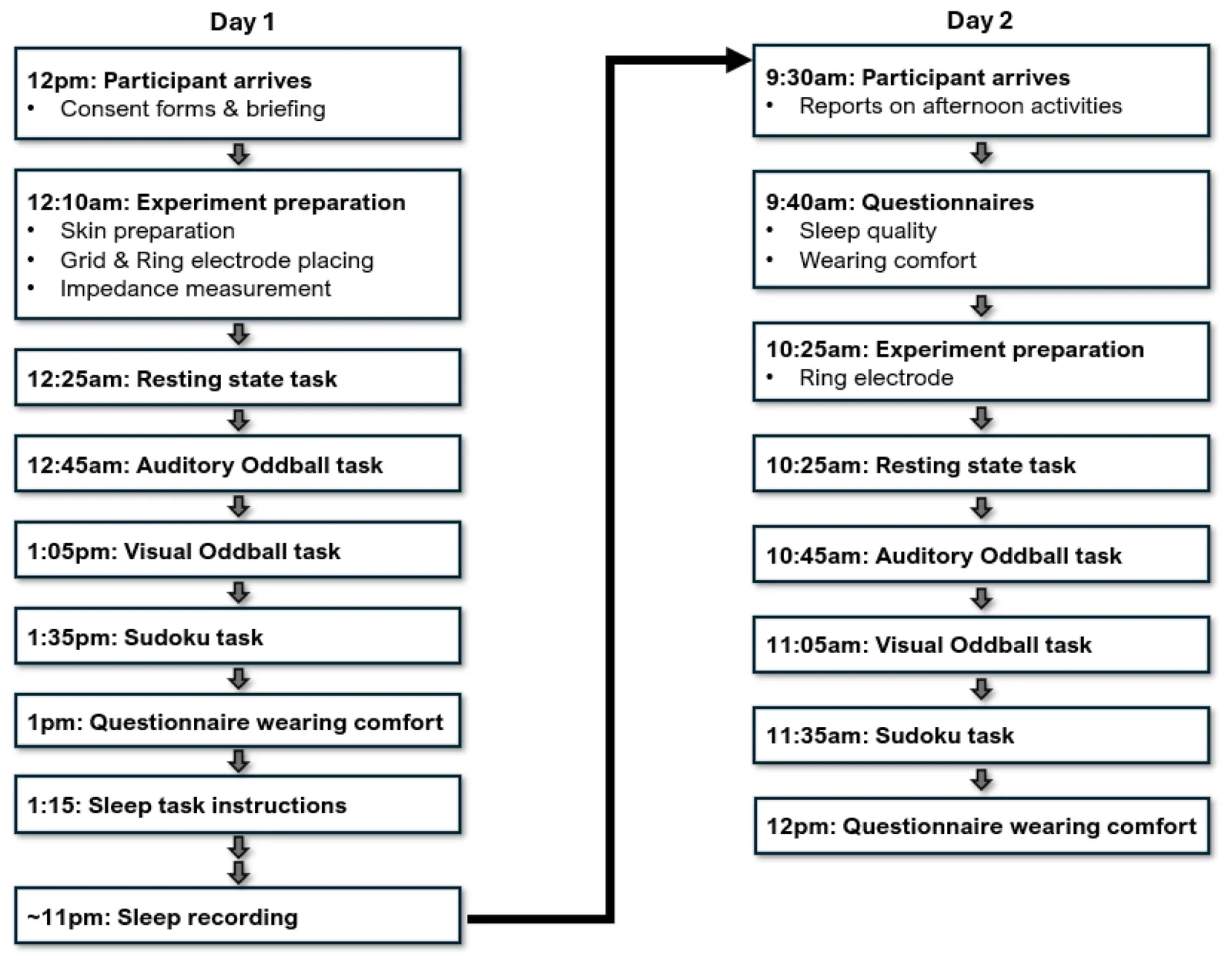
Flow chart of experimental tasks with approximate start times.

Each experimental task was performed twice over the 24 h period (see Figure 4). However, as the aim of the study was to reach 24 h, we were unable to record at the same time on both days. Therefore, tasks were performed in the early afternoon on the first day and in the morning on the second day. The tasks included both auditory and visual oddball tasks, followed by a resting state recording, and then a Sudoku task. The order of the two oddball tasks (auditory and visual) was counterbalanced across participants. Participants performed the sleep recordings at their own homes.

#### 2.5.1. Auditory Oddball Task

The auditory oddball task was programmed in Python 3.8.2 using PsychoPy (version 2025.1.1) [15] and PsychToolbox (version 3.0.19.14) [16] packages. During the task, participants sat two meters away from the speakers and looked at a fixation cross on the wall. The 12 min task consisted of a set of 800 tones. These included ‘standard’ frequent higher-pitch (886 Hz) and ‘deviant’ (17% or 136) infrequent lower pitch (443 Hz) tones. Tones lasted 62 ms, with 10 ms ramp-up and down and were separated by an inter-stimulus interval of 850 ± 50 ms. Tones were played in 4 three-minute blocks, and participants were asked to count the number of standard tones and report the number to the experimenter at the end of each block. Stimulus order was pseudorandomized to avoid having two deviant stimuli in a row or too close in sequence, with the same order for all participants and both days. The order of the two oddball tasks (auditory and visual) was counterbalanced across participants.

#### 2.5.2. Visual Oddball Task

**Figure 5:**
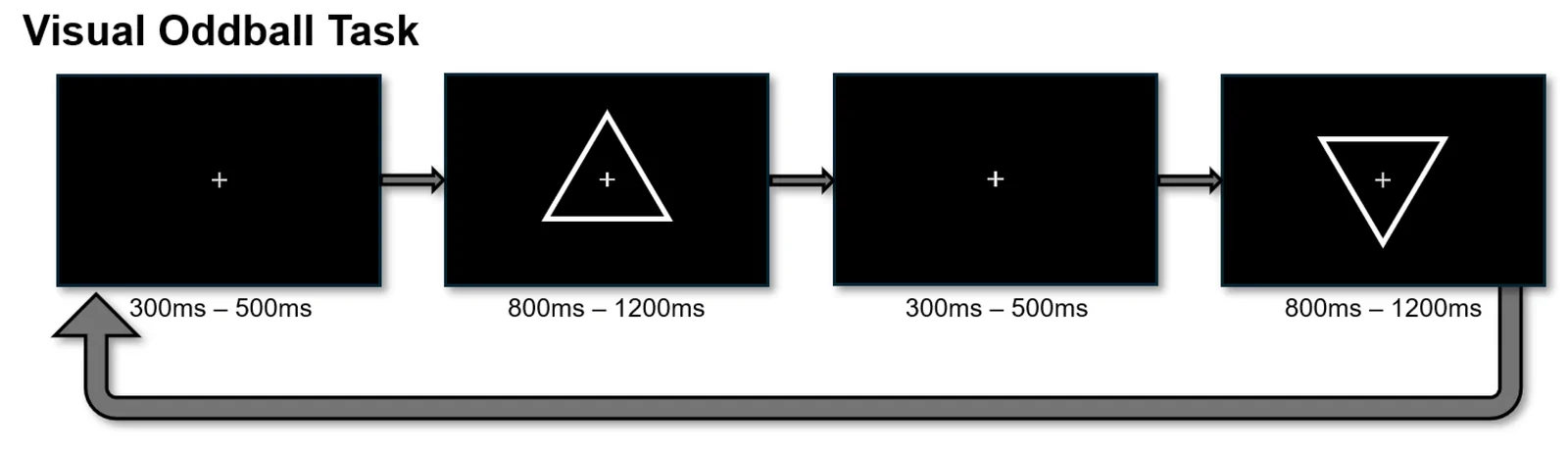
Pictorial representation of the visual oddball task. The triangle upwards was the deviant stimulus and the triangle downwards was the standard stimulus.

The visual oddball paradigm was programmed in Python 3.8.2 with PsychoPy (version 2025.1.1) [15] and based on a github repository [17], and Li et al. [18]. Oddball timing was adjusted to be similar to Krigolson et al. [19], to have greater variability and reduce the predictability of the stimuli, and also to compare the waveforms between the two studies. During the task, participants sat 1 m away from a computer monitor and looked at a fixation cross in the center of the screen. Participants then watched a series of white triangles appear around the fixation cross. The ‘deviant’ stimulus was a triangle pointing upwards, while the ‘standard’ stimulus was a triangle pointing downwards (see Figure 5). The triangles were equilateral with each side being 8.8 cm long (visual angle of 5.04°) on the screen. The fixation cross was 0.8 cm (0.46°) tall and wide on the screen. Between triangle appearances, participants saw only the fixation cross for 300–500 ms, while the triangles would appear for 800–1200 ms. The task included 514 triangles (86 or 17% deviants), making it last approximately 12 min, split into four three-minute blocks. Participants were asked to silently count the deviant upside-down triangles and report this number at the end of the trial. Stimulus order was pseudorandomized to avoid having two deviant stimuli in a row or too close in sequence, with the same order for all participants and both days.

#### 2.5.3. Resting State Task

The resting state task was programmed in Python 3.8.2 with PsychoPy (version 2025.1.1) [15]. The task included verbal instructions for the participant to sit still with their eyes open for two minutes, and closed for two minutes subsequently. During the eyes-open condition, participants stared at a fixation cross on a computer screen. The order of the eyes-closed and eyes-open conditions was counterbalanced within participants between day 1 and 2.

#### 2.5.4. Sudoku Task

The Sudoku task was programmed in Python 3.8.2 based on an online repository [20]. The task consisted of two 15 min Sudoku blocks, one which was ‘easy’ and another which was ‘difficult’, counterbalanced across participants and between days one and two. Sudoku puzzles were presented as an interactive GUI with 9 × 9 squares, with 9 boxes of 3 × 3 within. The ‘easy’ Sudoku blocks left approximately 20% of the squares empty, while the difficult block left approximately 65% of squares empty, sometimes filling in a few extra squares to ensure a valid solution. Participants would work on Sudokus continuously throughout the 15 min period, receiving feedback when all squares were filled in and then pressing a key to start a new puzzle.

#### 2.5.5. Sleep

For the data collection during sleep, participants took home the tablet computer, with a set of instructions, and also received a verbal description of how to begin collecting the data before going to sleep. Participants started the recording right before going to bed and disconnected again in the morning. Participants were asked not to shower in the evening or morning, in order to avoid any moisture damage to the miniaturized amplifier.

### 2.6. EEG Preprocessing

EEG preprocessing was performed in Python 3.11, using various external libraries including MNE (version 1.9.0) [21], NumPy (version 2.2.3) [22], pyprep (version 0.4.3) [23], csv (version 1.0), Matplotlib (version 3.10.1) [24], and Matlab R2022 (MathWorks Inc., Natick, MA, USA, [25]).

#### 2.6.1. Impedances

Impedances were recorded at 62.5 Hz in parallel to EEG recording. The values are provided from the amplifier as separate LSL stream sampled at approximately 1 Hz alongside the EEG data. For each of the active tasks (resting state, audio and visual oddball, Sudoku), timepoints between the ring and trEEGrid patch data were first equalized temporally to the beginning and end of the tasks, and then averaged over each channel for each participant and day. For the sleep impedance data (trEEGrid patch only), impedances were averaged over the first 2 h and the last 2 h for each participant and channel.

#### 2.6.2. Wearing and Recording Time

Wearing time was derived by taking the difference between the first and last storing time point of the xdf data file with the addition of the recording time of the first data file. The true wearing time was slightly longer, as the trEEGrid patch was applied 15–30 min before the start of the first recording. The total recording time is given as the sum of all recording durations for the participant over the two days.

#### 2.6.3. Oddball Tasks

First, ring (Fpz) and trEEGrid patch data were temporally equalized to the same start and end timepoints. trEEGrid patch channel locations were then spatially approximated to the standardized locations EOGh, EOGv, EOGd, Fpz_M2, C4_M2, P4_M2, O2_M2, EMG (see Figure 3), similar to previous studies using this montage [7,8]. Each stream was then bandpass filtered using 0.1 to 20 Hz finite impulse response filter (FIR), excluding also the noise from impedance measurement at 62.5 Hz. As LSL recorded the data for the Ring and trEEGrid patch with slightly differing nominal sampling rates, the data was then resampled (using mne.filter.resample() with a boxcar windowed fast-Fourier transform) to 250 Hz for each, to equalize the number of datapoints of the two streams and merged them into one dataset (MNE object). Bad channels were identified manually, by looking for excessive noise and signs of disconnection over significant periods in the data, and validated using the pyprep.find_noisy_channels() function. Data were then epoched from −200 ms to 1 s around the oddball stimuli, and baseline corrected with the pre-stimulus baseline period (−200 ms to 0 s). Epochs for all channels were rejected if the maximum peak-to-peak signal amplitude for any of the EEG channels (Fpz (ring), Fpz_M2, C4_M2, P4_M2, O2_M2) that exceeded 100 µV. For the P3 analyses, the remaining trials were then equalized between deviants and standards by removing a random permutation of trials from the standard stimuli to match the number of deviant stimuli. N1 analyses used only standards, and therefore did not require trial equalization. Then epochs were averaged over standard and deviant epochs for each participant, and the mean amplitude over the time windows for the N1 (80–120 ms; auditory task Fpz and Fpz_M2) and P3 (auditory: 286–386 ms, visual: 336–436 ms at Cz_M2) were exported for statistical analysis. ERP component time windows were determined according to peak amplitudes.

#### 2.6.4. Signal-to-Noise Ratio

SNR was computed on the N1 ERPs of standard trials during the auditory oddball task, to directly compare the ring (Fpz) and trEEGrid (FPz_M2). After computing the N1 ERPs as above, we then created two averaged ERPs per participant and sensor out of all the odd-numbered trials and another out of the even-numbered trials. We then took the mean of the two ERPs (i.e., signal) and absolute difference (i.e., noise) between the odd and even ERPs (see [26,27,28,29,30]). The SNR was then derived by dividing the absolute value of the mean signal by the mean noise within the N1 time window (80–120 ms) for each participant. This method allows for an estimate of noise within the same time window as the signal. SNR was then compared as an average value for each participant between conditions. To further investigate the number of trials required for each device to get to the maximum SNR, we plotted the SNR with trial numbers increasing incrementally by 2 (one even and one odd trial) and taking 100 trial permutations for each number of trials.

#### 2.6.5. Resting State

Resting state followed the same pipeline as the oddball ERPs until epoching, with the exception of the bandpass filter being 0.1 to 30 Hz finite infinite response filter (FIR). After merging the datasets and marking bad channels, 10 s regular epochs were created within the eyes-open and eyes-closed blocks. Power spectrum density was then computed using the multitaper method in the compute_psd() function in MNE, and was log transferred to decibel scale. Alpha power range was based on the mean peak frequency over all participants and days ±1.75 Hz. Power values were then averaged over the frequency range for each condition for each participant and day at channel O2_M2.

#### 2.6.6. Sudoku Task

Sudoku preprocessing also used the bandpass 0.1–30 Hz FIR filter. Additionally, to clean the EEG data Artifact-Subspace-Reconstruction (ASR; asrpy; 20 standard deviation threshold) was used after merging ring and trEEGrid patch data, to account for eyeblinks and free movement in this task [31]. Similar to resting state, 10 s epochs were taken in each block, and epochs were removed if any part of the epoch included time between finishing a puzzle and starting a new one, as well as if the peak-to-peak signal amplitude exceeded 500 μV. We used the multitaper method in the compute_psd() function in MNE, and PSD values were then log transferred and averaged for each participant, with mean theta (4–8 Hz) and low beta (13–20 Hz) power exported for statistical analysis. Frequency ranges were determined by previous literature [8,32,33,34].

To compare with resting state for low beta and theta power, eyes open resting state data from the resting state task was used as a baseline. This task data was processed again with all the same processing steps including ASR (20 SD) with mean theta and low beta powers exported, in order to be comparable with Sudoku task data.

#### 2.6.7. Sleep

Sleep EEG files were approximated spatially, then converted to EDF format and sent to a sleep staging expert for sleep staging according to AASM criteria using DOMINO software (version 3.1 ©SOMNOMEDICS, Randersacker, Germany).

### 2.7. Statistical Analysis

Statistical analyses were performed using JASP (version 0.95.1; [35]). EEG Measures included impedance within each day task and during sleep, signal to noise ratio, auditory N1 amplitude, auditory P3 amplitude, visual P3 amplitude, resting state alpha power, and beta and theta power during the Sudoku task. Additionally, questionnaire responses to the sleep quality and wearing comfort questionnaires are plotted.

For the task impedance, impedance values were averaged over the whole task for each participant at patch channel R1, and submitted to a repeated measures ANOVA with 2 factors: day and task. For sleep impedance, patch channel R1 was compared in a paired-samples *t*-test for data averaged over the first two hours and last two hours. For both the SNR and N1 amplitude, a repeated measures ANOVA was computed with two factors: day (1 and 2) and electrode (Fpz and Fpz_M2). For both the auditory and visual P3 amplitude, a repeated measures ANOVA was computed with the two factors of day and stimulus type (standards vs. deviants). A repeated measures ANOVA was also computed for resting state alpha power with the factors being day and eyes (open vs. closed). For the Sudoku, low beta and theta power analysis, theta power from the sudoku as well as from the eyes-open resting state were submitted to repeated measures ANOVAs with the three factors: electrode (Fpz_M2 vs. Fpz), day, and difficulty level (rest, difficult, easy). For ANOVA tests, degrees of freedom and *p*-values are reported using the Greenhouse–Geisser (GG) correction, when applicable. Effect sizes for *p* < 0.05 ANOVA effects are reported using partial eta squared (η_p_^2^). In the case of multiple *t*-tests, *p* values are corrected for multiple corrections using the Bonferroni correction [36], and significant *t*-tests are reported with effect sizes in the form of Cohen’s *d*.

## 3. Results

For all EEG recordings, one participant was excluded due to technical issues probably due to a damaged connector on the trEEGrid patch for the connection to the amplifier that occurred at the end of day 1 (S09). Due to the trEEGrid’s reduced density, channel interpolation in cases of bad channels is not possible. Therefore, participants with excessively noisy or disconnected channels were simply removed from the affected analyses (see Table 1). For impedance analyses during tasks, the data of nine subjects are used. For one participant the night recording was not completed due to an unplanned computer update of the operation system on the recording laptop (S07). Additionally, due to disconnected R1 channels overnight in S04, the data of seven participants were used for sleep impedance analyses. Sleep staging used the data of eight participants. Also, due to connection issues in R1 (Fpz_M2) for S04, the data of eight participants were used for SNR and N1 analyses. The auditory P3 analysis used the data of nine participants, and the visual P3 analysis used the data of eight participants due to a connection issue with C4_M2 in S05 during this task. Resting state alpha power used the data of eight participants due to issues with O2_M2 in S04. The Sudoku task analysis included eight participants, due to connection issues with Fpz_M2 for S04 during the resting state task.

### 3.1. Signal Quality

In Table 1 the trEEGrid patch wearing time is listed for all participants, with a range between 20.98 h and 23.67 h. EEG and impedance data were recorded in the controlled scenarios during day and night measurements, listed here as recording time between 3.55 h and 10.74 h. The low value of S07 is due to the missing night measurement.

**Figure 6:**
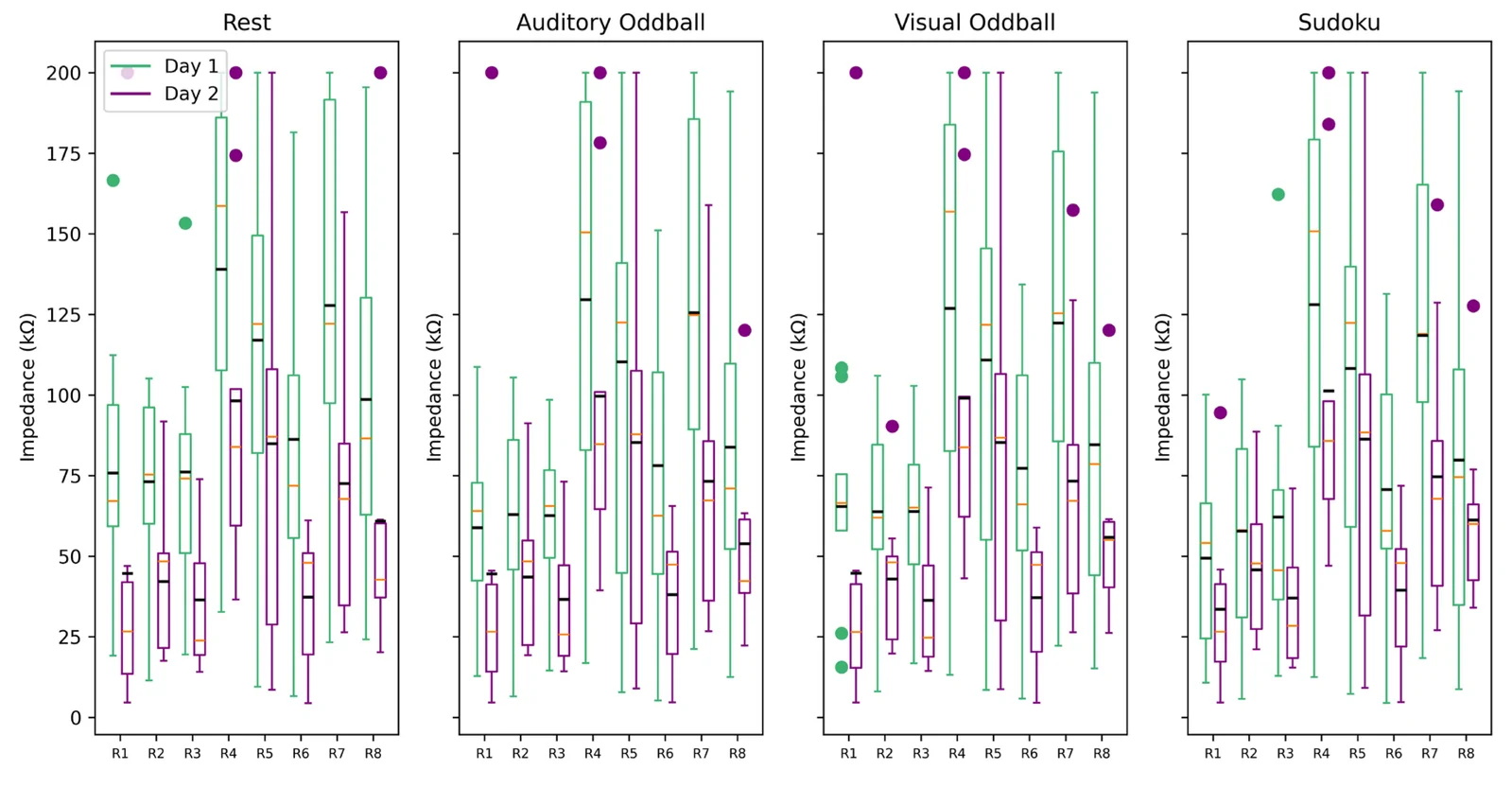
Boxplots of mean impedance over all participants for each task and each day. Grid channels are labeled R1–R8. Orange horizontal lines indicate the median, and black lines indicate the mean for each condition. Boxes indicate the first and third quartiles, while whiskers indicate the minimum and maximum, excluding outliers, which are depicted by dots.

Figure 6 depicts impedance boxplots for each task and day, over all participants. The box plots show mean impedance values between 49.4 kOhm (Sudoku, R1) and 139.1 kOhm (Resting task, R4) on day 1 (afternoon), and between 33.6 kOhm (Sudoku, R1) and 99.7 kOhm (Auditory Oddball, R4) on day 2 (morning), as well as a tendency to have overall lower impedance values on day 2 than day 1. Analyses of mean impedance values of patch electrode R1 for each task and day showed a significant effect of day (F(1, 8) = 6.37, *p* = 0.036, η_p_^2^ = 0.44), but no effect of task (F(3, 8) = 1.97, *p* = 0.19), or interaction (F(3, 8) = 4.43, *p* = 0.54). Ring electrodes had very low impedance, as expected, for all tasks in day 1 (mean impedance over tasks = 2.62 kOhm, and day 2 (mean = 2.42 kOhm).

For visualization purposes in Figure 6, we set the outlier threshold to 200 kOhm. The percentage of data exceeding this threshold is shown in Table 2 for each participant and each channel. For two participants (S07, S08) channel R5 has a high number of outliers with nearly half (S08: 49.30%) or even nearly all impedance values (S07: 99.73%) above 200 kOhm. For participant S07, channel R5 started at approximately 350 kOhm and ended at approximately 290 kOhm. For participant S08, channel R5 started with approximately 425 kOhm, around 625 kOhm in the morning of the second day and ended with approximately 580 kOhm.

**Figure 7:**
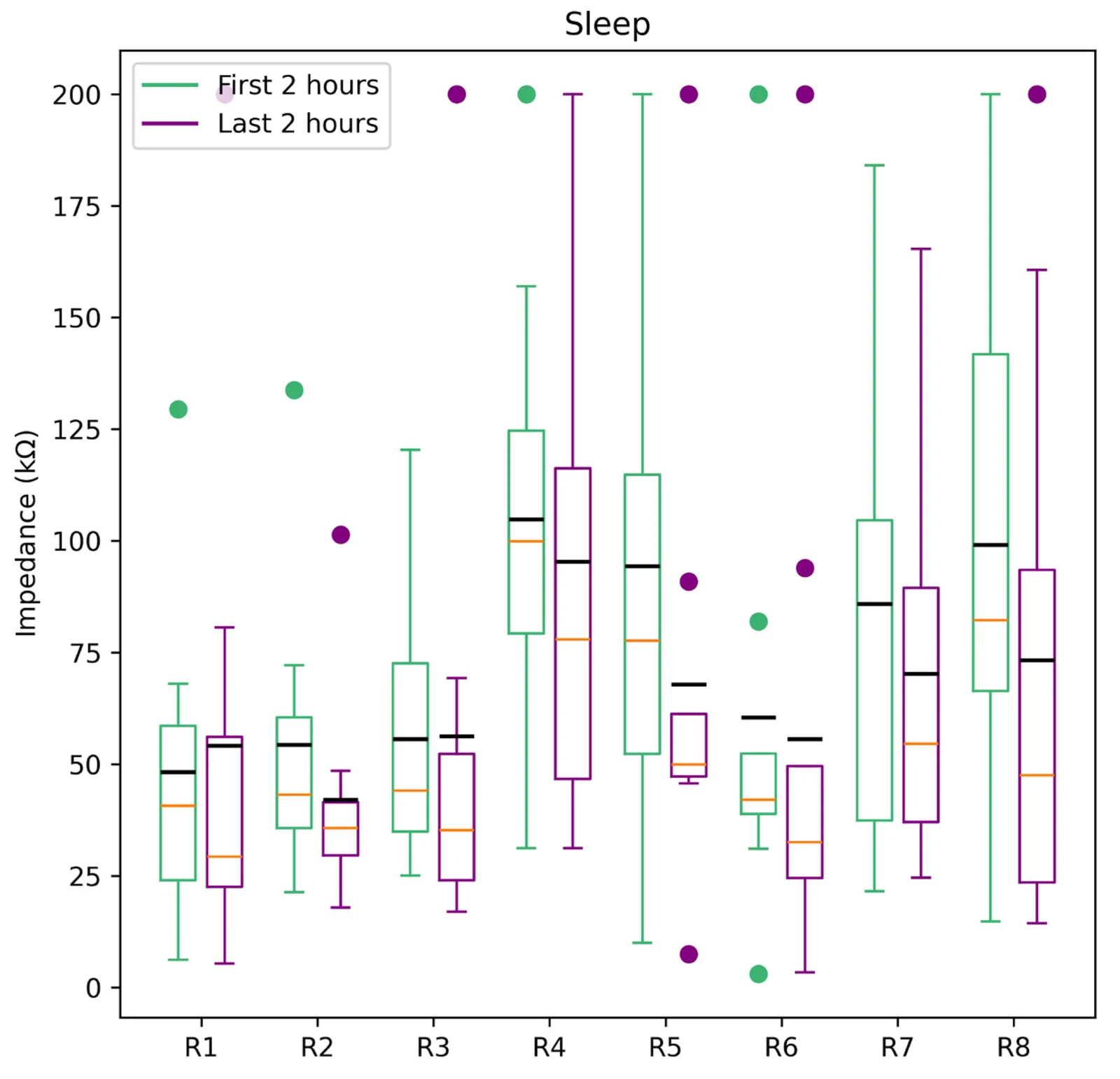
Boxplots of mean impedance during sleep over all participants for the first and last 2 h of each sleep recording. Orange horizontal lines indicate the median, and black lines indicate the mean for each condition. Boxes indicate the first and third quartiles, while whiskers indicate the minimum and maximum, excluding outliers, which are depicted with dots.

Figure 7 depicts boxplots of impedance values during sleep recordings, averaged over the first and last two hours of the recordings. The impedance values range from 48.2 kOhm (R1) to 104.8 kOhm (R4) in the first two hours and from 42.0 (R2) to 95.3 kOhm (R4) in the last two hours. For the sleep data, we found no significant difference between the first and last 2 h in R1 (*t*(6) = 0.23, *p* = 0.83).

**Figure 8:**
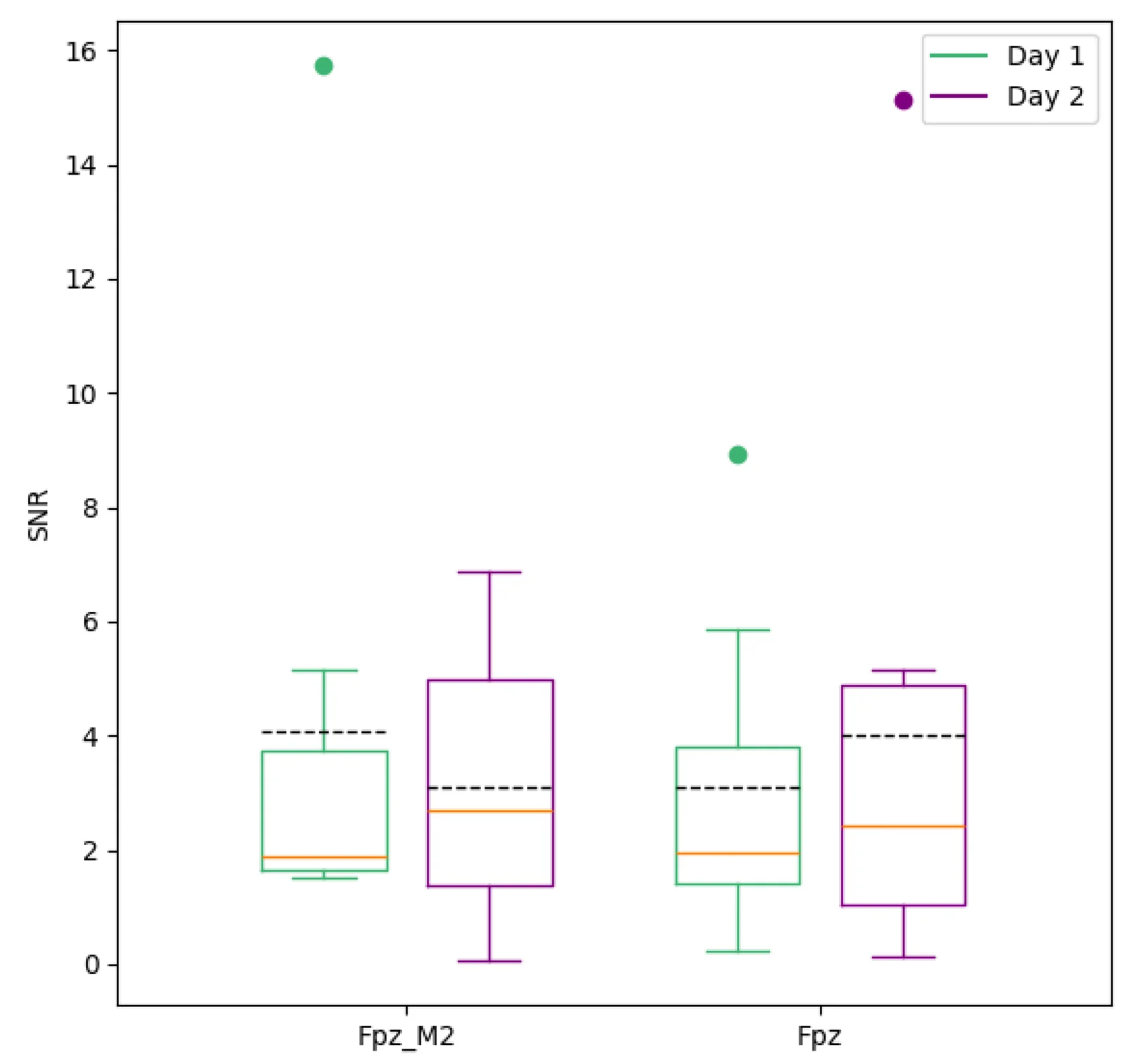
Boxplots of mean signal-to-noise ratio (SNR) for all participants during the N1 task, for both the Fpz_M2 and Fpz (ring) channels on both days. Orange horizontal lines indicate the median, and black dashed lines indicate the mean for each condition. Boxes indicate the first and third quartiles, while whiskers indicate the minimum and maximum, excluding outliers, which are depicted with dots.

**Figure 9:**
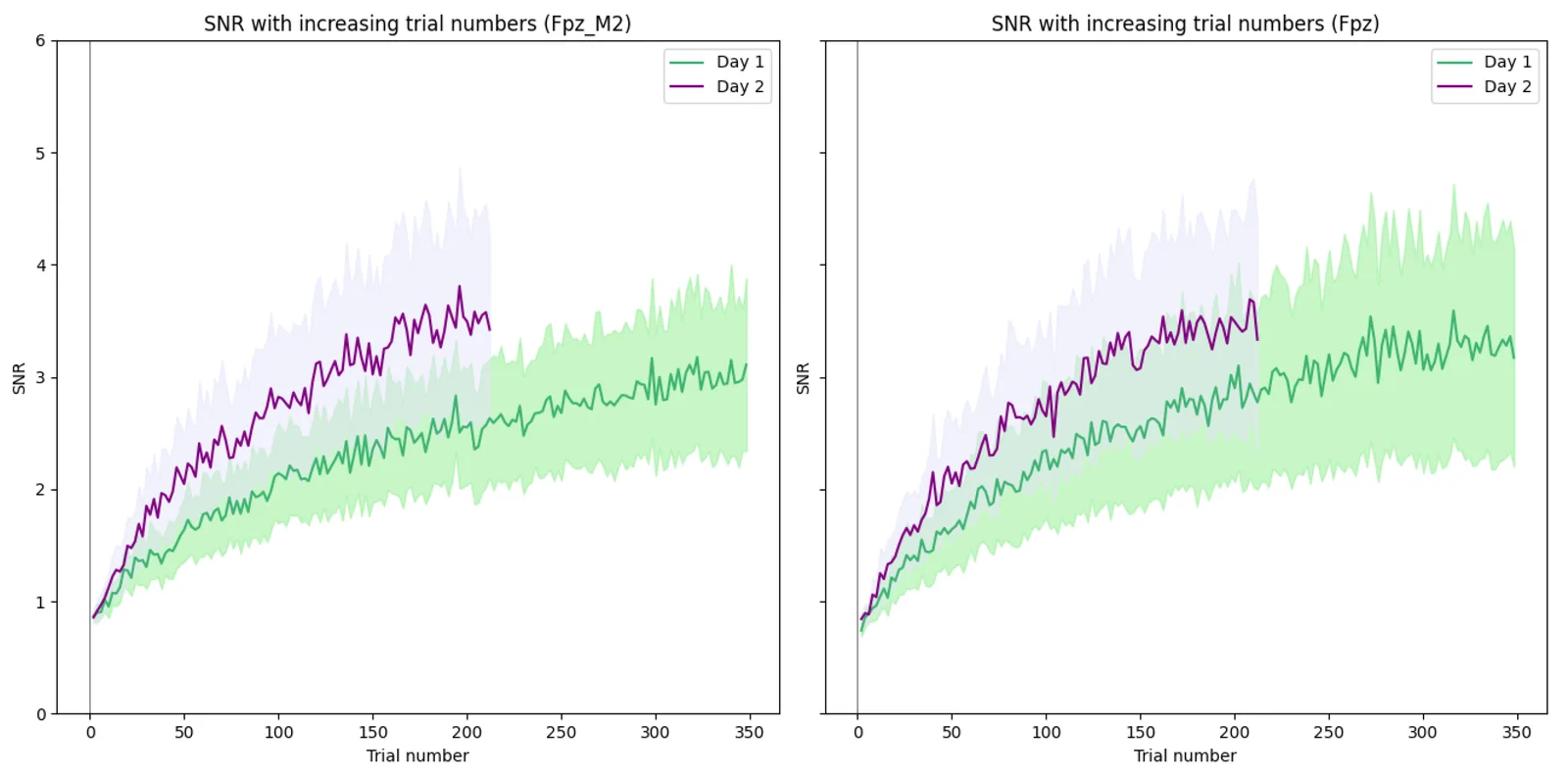
SNR computed with increasing trial numbers, with 100 trial permutations for each number of trials. (**Left**): Fpz_M2 and (**right**): Fpz. Shaded areas indicate the standard error of the mean.

Figure 8 depicts boxplots of SNR for the auditory oddball N1 for the Fpz_M2 and ring (Fpz) electrodes. Evident from the plots is a larger variance in day 2, but little difference between the two electrodes. Here we found no significant differences for the day (Means: Fpz_M2, day 1 = 4.09; Fpz_M2, day 2 = 3.09; Fpz, day 1 = 3.09; Fpz, day 2 = 4.01; F(1, 7) = 0.00065, *p* = 0.98) or electrode (F(1, 7) = 0.001, *p* = 0.98), and no interaction (F(1, 7) = 1.51, *p* = 0.26). Figure 9 depicts the change in SNR with increasing trial numbers, each with 100 permutations. Apparent from the plots is a slightly steeper slope and higher SNR values, but less trials remaining after artifact correction on day 2 (morning; mean = 512, SD = 125.2, min = 212) compared to day 1 (afternoon; mean = 543, SD = 88.2, min = 348) with all conditions plateauing to an SNR near 3. The trEEGrid Fpz_M2 shows a similar curve compared to the ring electrode Fpz. The Fpz SNR curve exhibits a large variance especially on day 1.

### 3.2. Results of the Experimental Tasks

#### 3.2.1. Auditory Oddball Task

**Figure 10:**
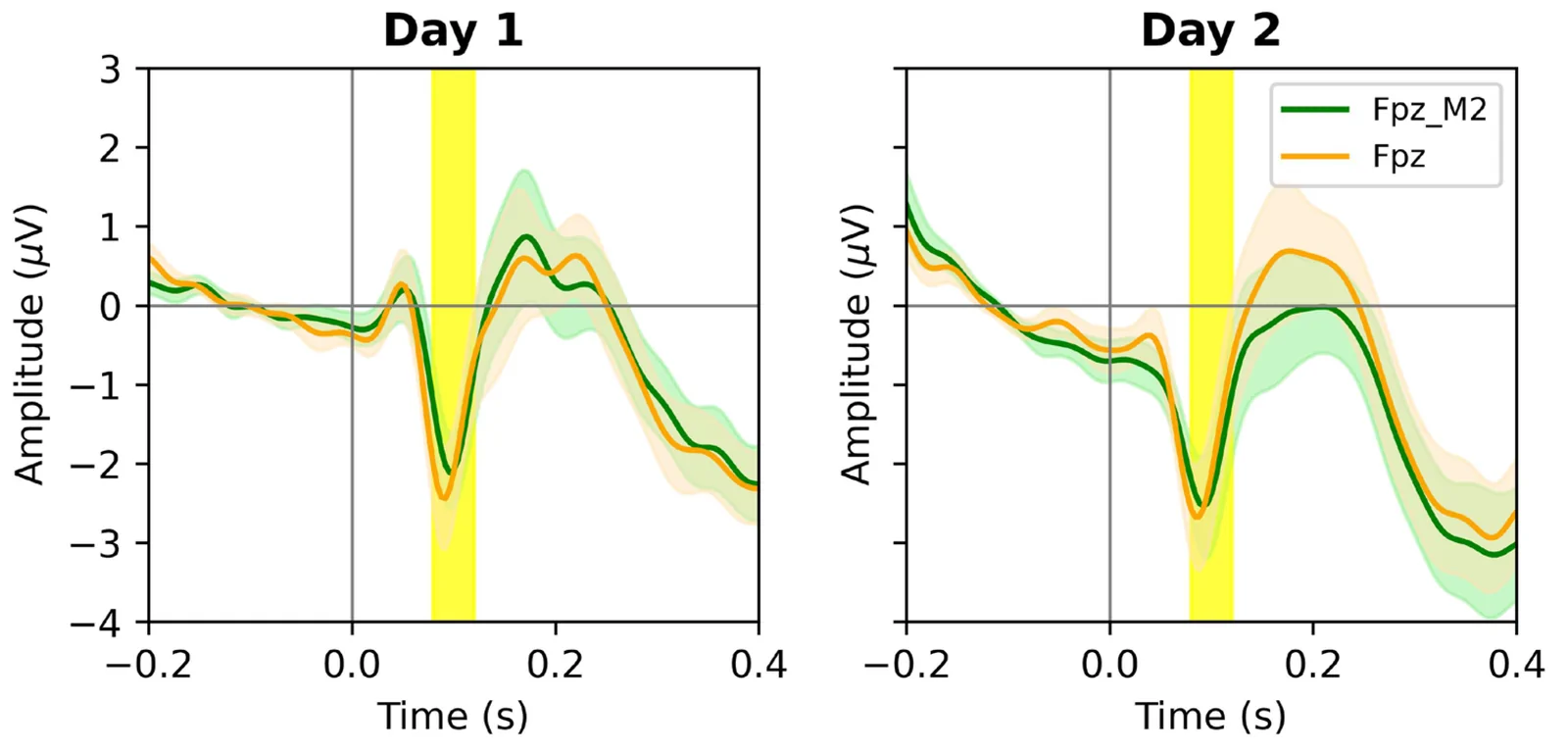
Auditory oddball task standard (non-deviant) ERPs for the frontal electrode Fpz_M2 and ring electrode (Fpz) on day 1 (**left**) and day 2 (**right**). The auditory N1 time window (0.08 to 0.12 s after stimulus onset (0.0 s)) is highlighted in yellow. Shaded areas indicate the standard error of the mean.

Figure 10 shows the auditory ERP for both frontal channels Fpz_M2 and the ring electrode (Fpz). Evident from the plots are similar N1 peak amplitudes around ~100 ms, around which we centered the N1 analysis. No significant N1 amplitude effects were found of the day (day 1 afternoon vs. day 2 morning; F(1, 7) = 0.46, *p* = 0.52) or electrode (F(1, 7) = 0.003, *p* = 0.96), and no interaction (F(1, 7) = 2.52, *p* = 0.16; (Means: Fpz_M2, day 1 = −1.64 μV; Fpz_M2, day 2 = −2.07 μV; Fpz, day 1 = −1.79 μV; Fpz, day 2 = −1.93 μV).

**Figure 11:**
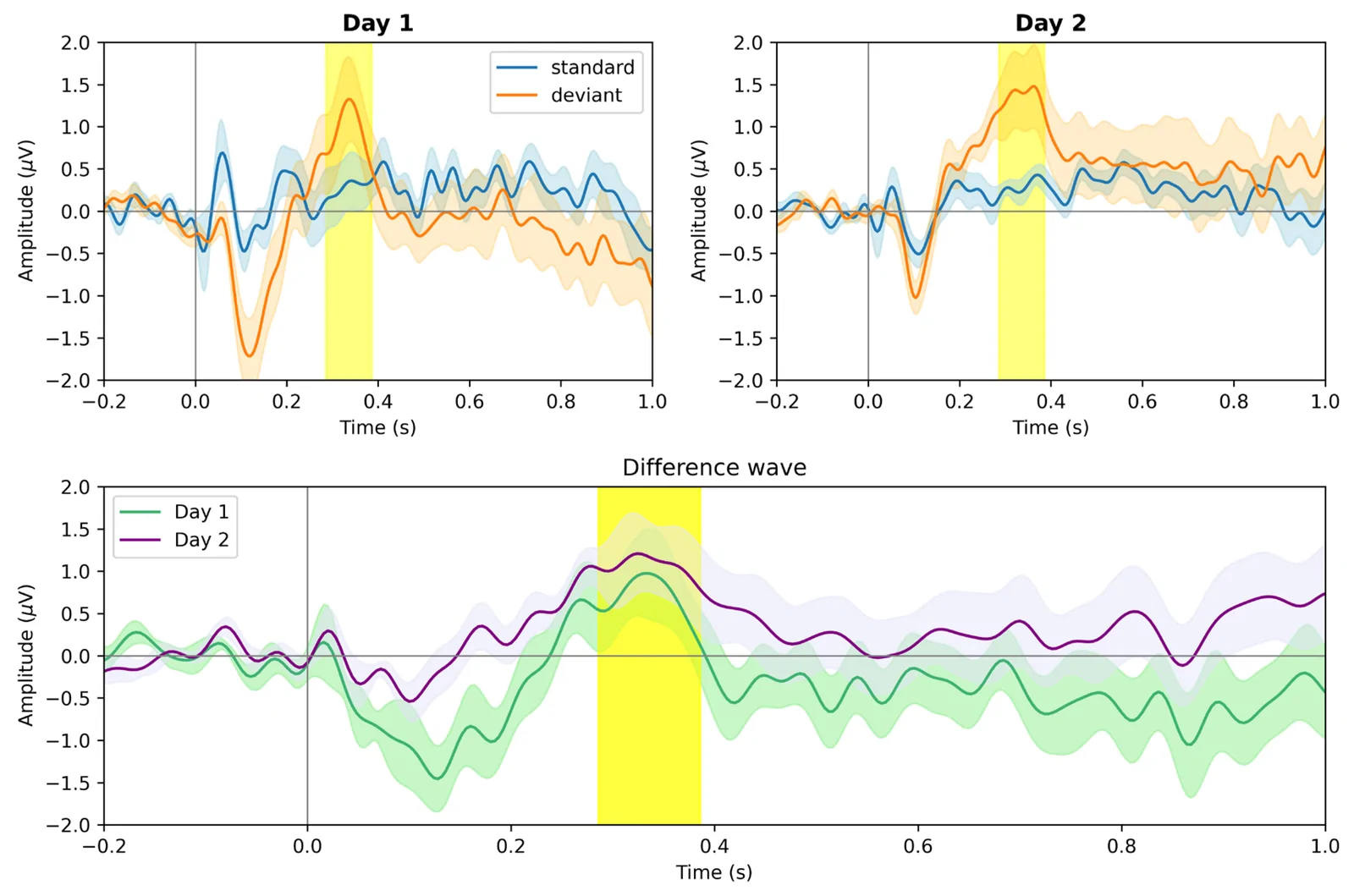
Auditory oddball task ERPs for the electrode C4_M2 on both days. The auditory P3 time window (0.29 to 0.39 s) is highlighted in yellow. Shaded areas indicate the standard error of the mean. (**Top**): standard and deviant stimuli for day 1 (**top left**) and day 2 (**top right**). (**Bottom**): P3 deviant-standard difference waves compared between days.

Figure 11 shows the auditory oddball P3 ERP component for standards and deviants in channel C4_M2 of the patch. This electrode was chosen due to the clear P3 peaks and more available subjects (*n* = 9), as two subjects had P4_M2 with impedance values larger than 200 kOhm on the second day, while this happened to only one participant for C4_M2. Evident from the plots is a larger peak around 336 ms in the deviants than the standards, around which we centered the analysis. Here we found a significant effect of stimulus type (standards vs. deviants; F(1, 8) = 7.4, *p* = 0.026, η_p_^2^ = 0.48) but no significant effect of the day (F(1, 8) = 0.54, *p* = 0.54) or interaction (F(1, 8) = 0.36, *p* = 0.57; Means: standard, day 1 = 0.28 μV; standards, day 2 = 0.30 μV; deviants, day 1 = 0.99 μV; deviants, day 2 = 1.37 μV).

#### 3.2.2. Visual Oddball Task

**Figure 12:**
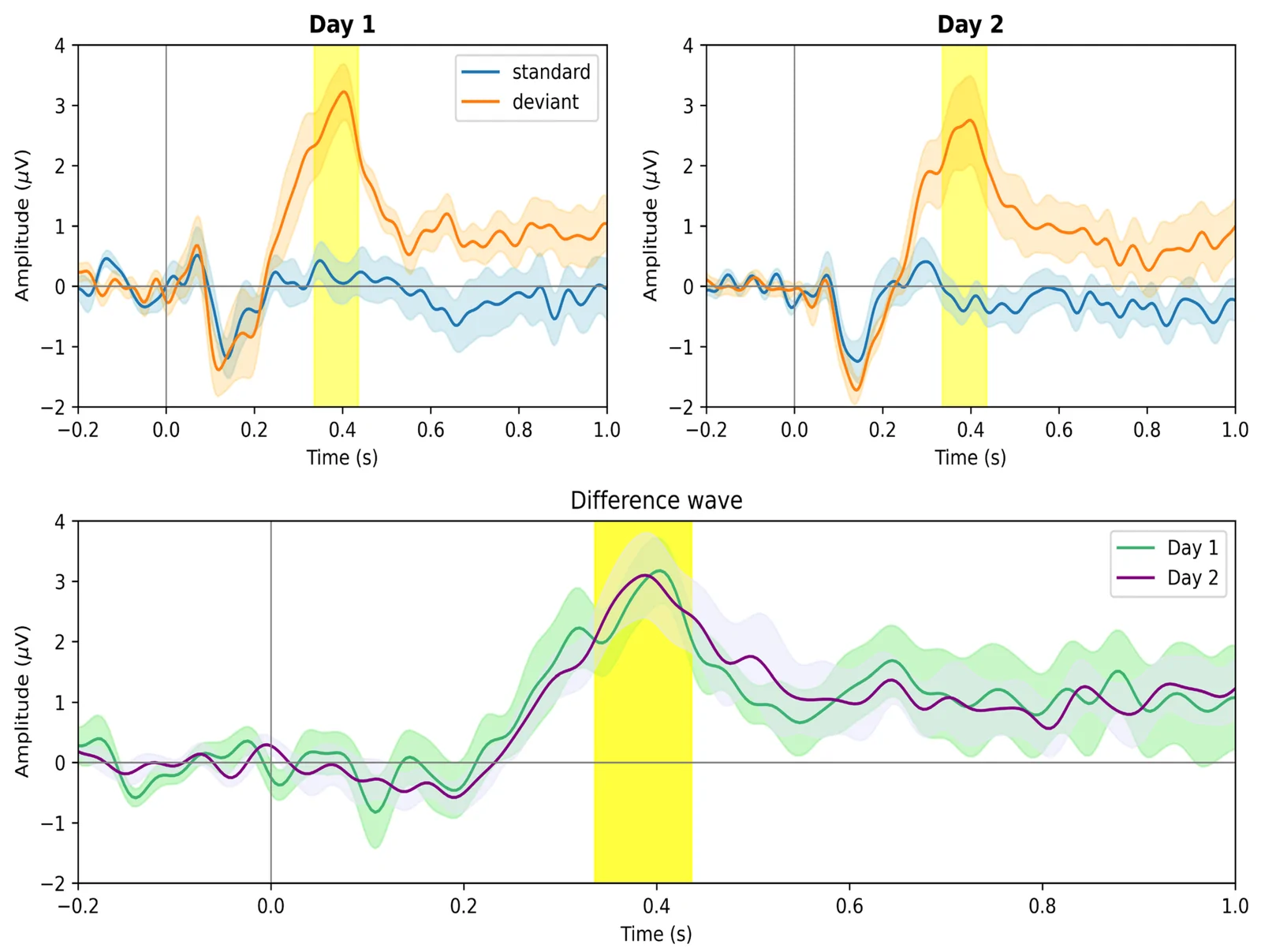
Visual oddball task ERPs for the electrode C4_M2 on both days. The visual P3 time window (0.34 to 0.44 s) is highlighted in yellow. (**Top**): Standard and deviant stimuli for day 1 (**top left**) and day 2 (**top right**). (**Bottom**): P3 Deviant-standards difference waves compared between days. Shaded areas indicate the standard error of the mean.

Figure 12 shows the visual oddball ERPs for the C4_M2 electrode on both days. Evident from the plots is a strong peak in the deviants around 386 ms after the stimuli. For P3 amplitude (Means: standards, day 1 = 0.20 μV; standards, day 2 = −0.25 μV; deviants, day 1 = 2.80 μV; deviants, day 2 = 2.48 μV), we found a significant effect of stimulus type (F(1, 7) = 22.26, *p* = 0.002, η_p_^2^ = 0.76), but no effect of day (F(1, 7) = 1.84, *p* = 0.22) or interaction (F(1, 7) = 0.117, *p* = 0.74).

#### 3.2.3. Resting State Task

**Figure 13:**
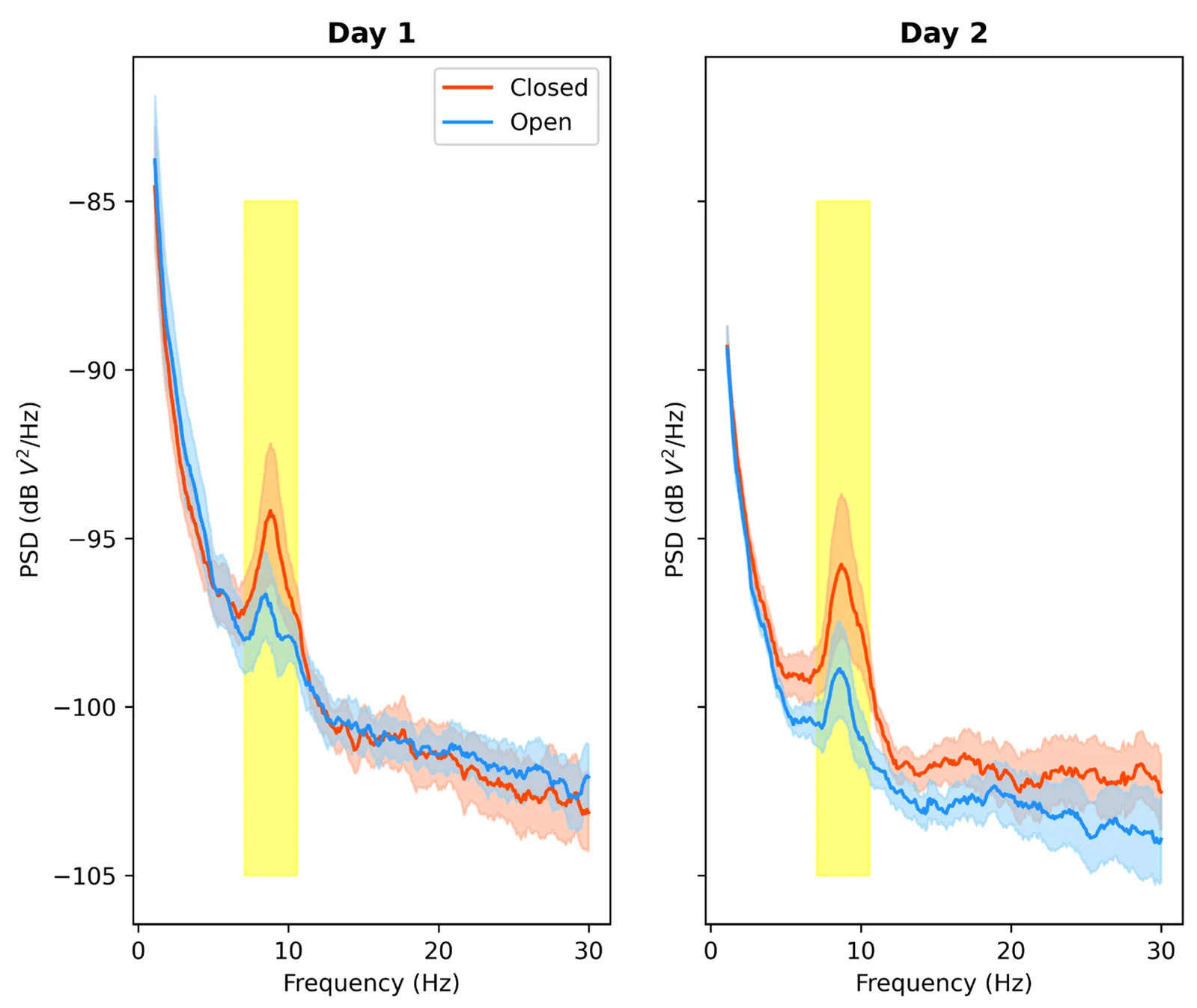
Resting state spectral activity during eyes open and eyes closed on day 1 (**left**) and day 2 (**right**) for channel O2_M2. Frequencies of interest are highlighted in yellow (7.07–10.57 Hz). Shaded areas indicate the standard error of the mean.

Figure 13 depicts the resting state spectral activity (power spectral density (PSD)) for channel O2_M2 during eyes closed and eyes open for both days. Evident from the plots is an alpha peak around 8.82 Hz, around which we centered the analysis (±1.75 Hz). Here we found a significant effect of alpha for eyes open/closed (F(1, 7) = 15.84, *p* = 0.005, η_p_^2^ = 0.69), with alpha power being higher when the participants’ eyes were closed (Means: open, day 1 = −97.31 V^2^/Hz, day 2 = −99.62 V^2^/Hz; closed, day 1 = −95.33 V^2^/Hz, day 2 = −96.67 V^2^/Hz). Additionally, we found a significant effect of the day (F(1, 7) = 24.0, *p* = 0.002, η_p_^2^ = 0.77), and an interaction (F(1, 7) = 10.13, *p* = 0.015, 0.59), driven by higher alpha power on day 1 (afternoon) than day 2 (morning), especially in the eyes open condition.

#### 3.2.4. Sudoku Task

**Figure 14:**
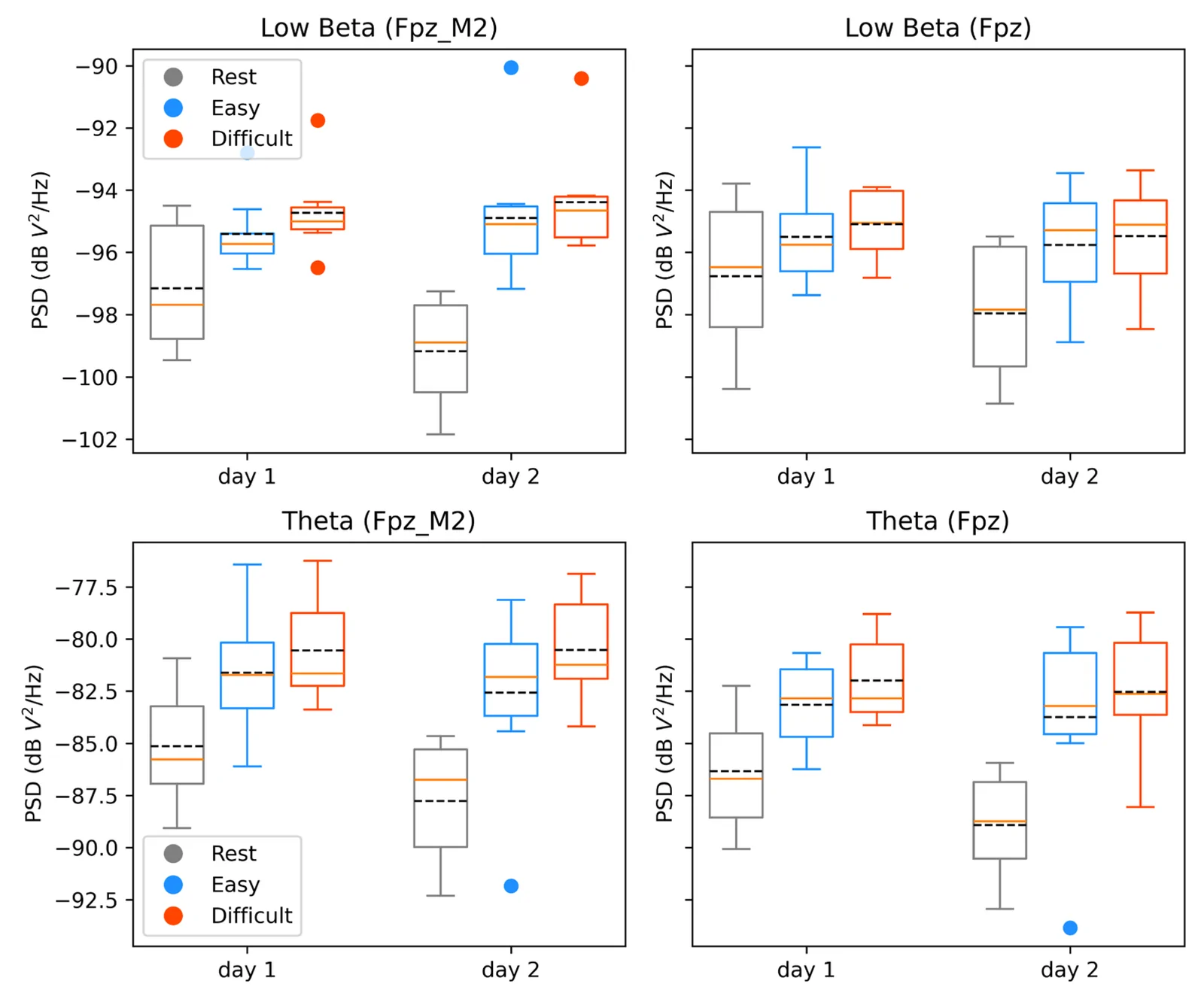
Spectral markers in the Sudoku task. (**Top**): Boxplots of low beta power during Sudoku task for channel Fpz_M2 and ring (Fpz) electrode. (**Bottom**): Boxplots of theta power during Sudoku task for channel Fpz_M2 and ring (Fpz) electrode. Orange horizontal lines indicate the median, and black dashed lines indicate the mean for each condition. Boxes indicate the first and third quartiles, while whiskers indicate the minimum and maximum, excluding outliers, which are depicted with dots.

This study looked at the theta (4–8 Hz), and low beta (13–20 Hz) power between resting state and the two levels of Sudoku (difficult and easy), both frontal electrodes and across both days. For frontal low beta power (13–20 Hz), we found a significant effect of level (F(2, 14) = 17.31, *p* = 0.004, η_p_^2^ = 0.71), but no effect of electrode (F(1, 7) = 0.094, *p* = 0.77), or day (F(1, 7) = 2.29, *p* = 0.17). We did however find an interaction effect between condition and electrode (F(2, 14) = 8.32, *p* = 0.018, η_p_^2^ = 0.54) and between condition and day (F(2, 14) = 5.79, *p* = 0.028, η_p_^2^ = 0.45) and no other interaction effects (all F’s < 3, all *p*’s > 0.1; means: rest: Fpz, day 1 = −96.77, Fpz, day 2 = −97.96; Fpz_M2, day 1 = −97.16, Fpz_M2, day 2 = −99.18; difficult: Fpz, day 1 = −95.10 V2/Hz, Fpz, day 2 = −95.48 V2/Hz; Fpz_M2, day 1 = −94.73 V2/Hz; Fpz_M2, day 2 = −94.39 V2/Hz; easy: Fpz, day 1 = −95.5 V2/Hz, Fpz, day 2 = −95.76 V2/Hz; Fpz_M2, day 1 = −95.41 V2/Hz; Fpz_M2, day 2 = −94.90 V2/Hz). Figure 14 (top) depicts low beta power from the Sudoku task at both Fpz_M2 and Fpz.

To investigate the interaction between difficulty level and day, we performed post hoc comparisons between days at each level, finding a significant difference in low beta in the resting state of day 1 (afternoon) and day 2 (morning; *t*(7) = 5.55, *p*_bonf_ < 0.001, *d* = 0.89), with no other significant differences (all *t*’s < 2, all *p*_bonf_’s > 0.2). This indicates that this interaction was driven by differences in the resting state between the two testing timepoints. Additionally to understand the difficulty level x electrode interaction, follow up comparisons showed at electrode Fpz that low beta power differed significantly (*t*(7) = 3.89, *p*_bonf_ = 0.02, *d* = 1.13) between rest and the difficult Sudoku condition. However, at Fpz, rest did not differ from the easy Sudoku condition (*t*(7) = 3.02, *p*_bonf_ = 0.06) nor did the easy and difficult Sudoku condition differ from each other (*t*(7) = 0.34, *p*_bonf_ = 0.17). On the other hand, at electrode Fpz_M2 all follow up comparisons reached significance. That is, rest differed significantly from easy Sudoku (*t*(7) = 1.65, *p*_bonf_ = 0.02, *d* = 1.65) and the difficult Sudoku condition (*t*(7) = 1.97, *p*_bonf_ = 0.008, *d* = 1.97) and the easy and difficult Sudoku levels also differed significantly from each other (*t*(7) = 5.51, *p*_bonf_ = 0.003, *d* = 0.32).

Figure 14 (bottom) depicts theta power from the Sudoku task at both Fpz_M2 and Fpz. For theta power, we found a significant effect of the level (F(2, 14) = 10.81, *p* = 0.008, η_p_^2^ = 0.61), electrode (F(1, 7) = 26.13, *p* = 0.001, η_p_^2^ = 0.79), and the day/time (F(1, 7) = 6.65, *p* = 0.037, η_p_^2^ = 0.49), but no interactions (all F’s < 2, all *p*_bonf_’s > 0.2; Means: rest: Fpz, day = −86.34, Fpz, day 2 = −88.91; Fpz_M2, day 1 = −85.14, Fpz_M2, day 2 = −87.76; difficult: Fpz, day 1 = −81.99 V2/Hz, Fpz, day 2 = −82.53 V2/Hz; Fpz_M2, day 1 = −80.54 V2/Hz; Fpz_M2, day 2 = −80.52 V2/Hz; easy: Fpz, day 1 = −83.15 V2/Hz, Fpz, day 2 = −83.74 V2/Hz; Fpz_M2, day 1 = −81.61 V2/Hz; Fpz_M2, day 2 = −82.57 V2/Hz).

#### 3.2.5. Sleep

**Figure 15:**
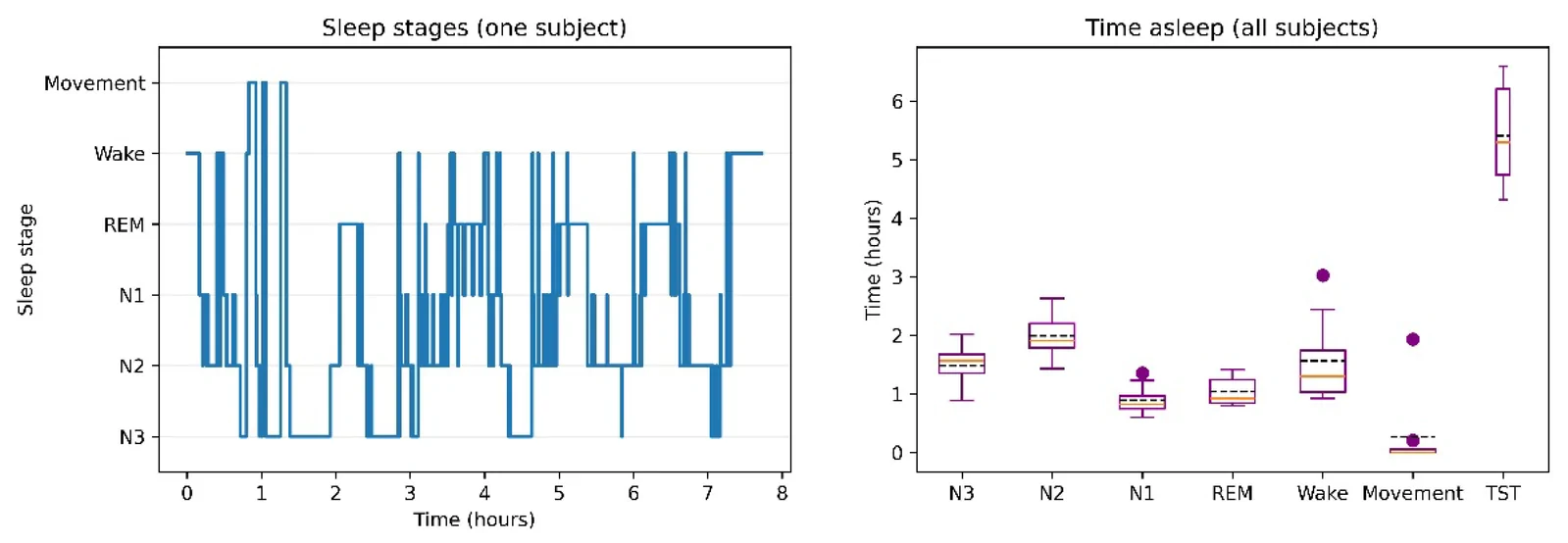
Sleep data. (**Left**): Hypnogram based on annotations by an expert scorer for one participant (S02). (**Right**): Boxplot of all sleep stages and total sleep time (TST) over all participants. Orange horizontal lines indicate the median, and black dashed lines indicate the mean for each condition. Boxes indicate the first and third quartiles, while whiskers indicate the minimum and maximum.

Sleep data for all participants, with the exception of the two who experienced technical problems during recording, were annotated by a sleep scorer. The sleep scorer has more than 15 years’ experience in annotating polysomnography data working in a sleep laboratory. Based on the spatial approximation, the trEEGrid data can also be analyzed visually by sleep scorers. A quality comparison regarding the approximation of electrode positions for sleep scoring was conducted in da Silva Souto et al. [6]. The mean total sleep time for all recorded participants was 5.41 (SD = 0.81; range = 4.3 to 6.6) hours. Due to the limited battery life, the study was not designed to investigate sleep duration; therefore, this aspect is not examined further here. Sleep metrics are shown in Figure 15.

### 3.3. Questionnaires

**Figure 16:**
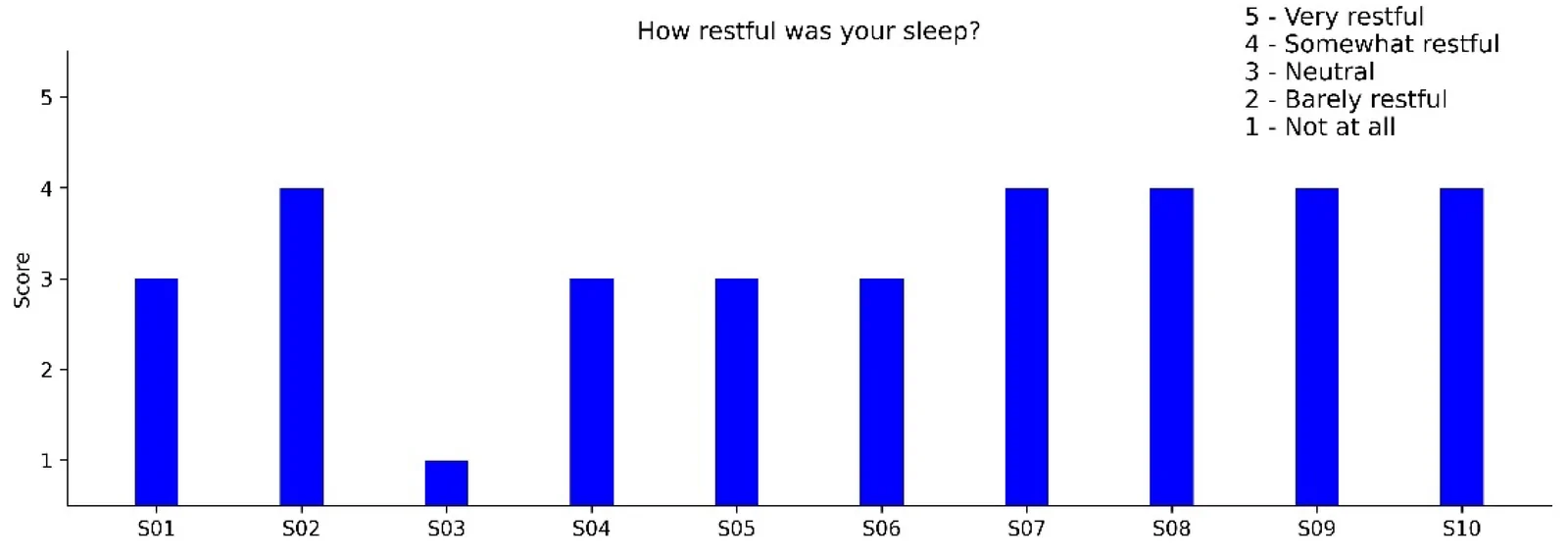
Sleep quality questionnaire results, reported on the morning of day 2.

Figure 16 shows self-reported answers to questionnaires about perceived sleep quality and wearing comfort. The mean score for sleep quality was 3.3 (SD = 0.9) out of 5, with 50% of participants reporting at least somewhat restful sleep. Participants rated wearing comfort on a Likert scale (1 = very poor to 5 = very good). For each time point of the wearing comfort assessment, eight participants rated wearing comfort as 5 (very good) and two participants rated wearing comfort as a 4 (good), with a mean rating of 4.8 (SD = 0.39). Additionally, participants rated whether they experienced specific irritations including itching, pressure, poking and annoyance with the trEEGrid patch and mini-amplifier on a Likert scale (1 = fully untrue, 5 = fully true), in which no ratings were over 3 (3 = partly true) on day 1 or day 2.

## 4. Discussion

In this study, participants wore trEEGrid patches with a mini amplifier prototype for nearly 24 h, completing four different cognitive tasks twice, as well as sleeping at their own homes. Impedance improved over time significantly when comparing tasks between day 1 and 2, with SNR values remaining stable between the two days. During cognitive tasks, in addition to the trEEGrid patch, EEG activity was also recorded with a ring electrode and mobile EEG amplifier for comparison of the trEEGrid patch to a conventional EEG recording method. Additionally, participants filled out questionnaires regarding their sleep quality and wearing comfort with the long-term EEG system. Results showed no significant differences in auditory N1 amplitude between the ring and trEEGrid patch channels, and both systems showed significant effects of difficulty level in low beta and theta power during the Sudoku task. Additionally, the trEEGrid patch was able to show typical P3 differences between standards and deviants in both auditory and visual oddball tasks, as well as a significant increase in alpha power while participants eyes were closed in resting state. Sleep data was successfully recorded in participants’ homes and annotated into sleep stages by a sleep-staging expert, demonstrating the feasibility of this system for home sleep monitoring.

### 4.1. Signal Quality

Impedance decreased significantly over time from day 1 (afternoon) to day 2 (morning) for patch R1 electrode in all tasks, with no effect of the task or interaction. This is similar to previous studies of impedance with other pre-applied materials such as hydrogel [9] (da Silva Souto et al., 2025). We did not find a significant change in impedance between the first and last 2 h of the sleep recording. However, impedance patterns can vary considerably over the course of the night, partly due to changes in sleeping position, including lying on the trEEGrid patch, cf. Supplementary Figure S2.

Impedance in the patch electrodes is generally higher than ring electrodes (all <~10 kOhm); however, no differences were found between the SNR of the Fpz ring electrode and the Fpz_M2 electrodes in this stationary setting without or with very little cable movements. Both systems had an SNR of 2:1 after ~150 good trials, continuously increasing with additional trials.

Most channels in the system were still viable at the end of the nearly 24 h study for most participants, which is a large improvement over conventional EEG. Given the small number of electrodes, it is not possible, or it is only to a very limited extent, to interpolate data from failed electrodes using the other electrodes in the trEEGrid system. It is important therefore to design the system for good signal quality and long-term wear in all channels. As shown in Table 2, R4 and R5 were the most problematic electrodes with the highest percentages of impedance outliers (>200 kOhm) over most subjects. Channel R5 is located near the eyebrow (see Figure 1) and may be more strongly affected by muscle activity, skin movement and hair in that area. The placement of this electrode will be reconsidered in the next iteration of the patch design. For channel R4, impedance exceeded the 200 kOhm threshold in 12–20% of the recorded data of participants. This channel is located in the lower part of the chin (see Figure 1) and is therefore subject to movement both when speaking and when chewing. In male participants (2 of the 3 subjects), facial hair can also make skin contact more difficult. Due to these issues reducing the number of viable participants for some measures, R4 was not used in the EEG analysis in the current study (i.e., P4_M2 replaced by O2_M2 for resting state and C4_M2 for P3 amplitude). This position will also be reconsidered in the next patch design iteration.

### 4.2. ERP Components

The auditory N1 in response to the standard trials in the auditory oddball task demonstrated a significant peak in our data, in both the patch and ring electrodes. We do not claim that the ring and patch electrodes measure the N1 equivalently, as the electrodes were in slightly different locations and we have not explicitly tested equivalence (e.g., with a Two One-sided *t*-test). However, clear N1 components can be observed in both the patch and ring electrodes with no significant difference in amplitude, demonstrating that either of these systems can be used for analyzing auditory responses. Additionally, no amplitude differences were found between day 1 (afternoon) and 2 (morning), and no interaction. This shows that changes in impedance, longer wearing time and time of day did not reduce the patch’s ability to record the auditory response, similar to our previous studies with pre-applied trEEGrid systems [9].

In both the auditory and visual oddball task a clear P3 component was detected at electrode C4_M2. Additionally, there was again no significant difference between day 1 (afternoon) and day 2 (morning). Our auditory P3 response is similar in morphology to previous studies using cEEGrids to analyze the oddball response [1,3]. cEEGrid studies often use an approximation which average and subtract electrodes above the ear to those below the ear, leading to a P3 response similar to what would be seen at Cz or Pz, albeit with a lower amplitude [3,37,38]. Our data shows a slightly smaller amplitude than this, likely due to our available electrodes behind the ear being closer together than those available in the cEEGrid, yet the difference was strong enough to clearly see the oddball response.

For the visual P3 response, our P3 morphology can be compared to the study by Krigolson et al. [19]. Notably, the trEEGrid patch P3 difference (deviants-standards) response is similar in amplitude and morphology to a similar task response captured with the MUSE consumer EEG and TP9/TP10 in an EEG cap [19]. This shows again that even with unconventional channel locations, well-known robust brain responses can be well-quantified using discrete long-term wearable reduced electrode systems.

### 4.3. Spectral Signals

In our resting state task, we found main effects for the eyes open/closed as well as for the day/time and an interaction between these effects. The main effect for eyes open/closed demonstrates that this system is sensitive enough to find a well-known effect of increased alpha power when eyes are closed at occipital channels [12], due to visual suppression [39].

The effect of day in both alpha, low beta and theta power might be related to the time of day being different between the two recordings. In order to limit total study participation time to a maximum of 24 h, recordings for day 1 took place in the afternoon while day 2 recordings took place in the morning. Therefore, this led to the limitation that we could not have both sessions at the same time of day. Previous studies have found that evening-type chronotype people had increased posterior channel alpha power in late afternoon sessions compared to morning sessions [40]. While our study did not take chronotype into account, it is possible that enough participants skewed towards ‘evening’ type to affect the overall alpha power average. The analysis of low beta during the Sudoku task relative to the resting state baseline condition revealed an interaction between condition and difficulty level. This interaction appears to be driven mostly by differences in the resting state condition, which could be expected due to the time difference. Resting state beta power has been shown to be higher in the afternoon than morning [41]. Additionally, the decrease in theta power from day 1 to day 2 in the Sudoku task could, similar to the alpha and beta results, be related to the time of day of the recording. Theta power has been shown to increase with time spent awake, especially in frontal areas [42,43]. Indeed, that we were able to show these effects with eight participants in both trEEGrid patch and ring electrodes demonstrates the sensitivity of our miniaturized system.

An ideal application for long-term unobtrusive EEG recording in daily life is to observe markers of cognitive workload. Previously we have been able to observe vigilance changes over time using both a trEEGrid and cap system during simulated driving [8] by observing changes in frontal beta and theta power. We observed that both frontal beta and theta were higher during the monotonous autonomous driving task compared to the more engaging manual driving, and also both increased over time. Frontal low beta (13–20 Hz) has been interpreted as a marker of mental effort [33], and frontal theta (4–8 Hz), has been shown to reflect mental workload [34,44].

The Sudoku task was exploratory but has the potential to be a useful and ecologically valid proxy measure of real-world workload in the future. Here we found that low beta power on frontal electrode sites was higher for Sudoku than the resting (baseline) condition and also higher for the difficult than easy Sudoku condition, on both days and for both electrodes (ring and trEEGrid patch). This demonstrates increased mental effort in the difficult condition, as beta activity has been associated with managing task-related workload, mental resources [45], and cognitive workload [46].

Wascher et al. [32] showed an observable increase in evoked theta activity during a Sudoku task compared to a resting state. Here we also found a significant effect of the difficulty level when comparing theta activity during our resting state baseline condition to easy and difficult Sudoku tasks. While solving the Sudoku puzzles theta was increased compared to rest, indicating increased cognitive control during the Sudoku task [32].

A significant theta difference between the ring and trEEGrid patch electrodes may indicate differences due to the electrode location and possible issues with the impact of ocular artifacts. The Fpz_M2 electrode was closer to the eyes, and also had higher impedance levels than the Fpz ring electrode. Therefore the increased theta power at the Fpz_M2 channel could possibly be due to larger eyeblink amplitudes at this channel. Eyeblinks can influence EEG activity in the theta band [47] especially in frontal electrodes. ASR was used to process the EEG data of this task, and has been shown to reduce ocular artifacts [31,48]. The ASR parameter of a 20 SD cutoff is on the lower end of the typical recommended cutoff range for ASR, which is 20–30 SD [49], as cutoffs lower than 10 SD could risk removing brain activity from the data [48]. However, it is possible that remaining ocular activity might have contributed to the recorded theta activity, leading to the difference between Fpz_M2 and Fpz. It is unlikely that blink activity caused the Sudoku difficulty level effects, because blink frequency tends to decrease during reading [50] and computer tasks [51] compared to passive tasks, while our theta results showed the opposite effect. Additionally, it is also unlikely that eyeblinks influenced the day-related effects on theta power, which as mentioned above, is likely due to the time of day during tasks (i.e., morning vs. afternoon). Studies of blink rate have demonstrated stable blink frequency between the morning and afternoon, with increases in the evening (i.e., after 8 pm [52]), and therefore would not make a difference during our recording times. In the future we would like to derive an adequate eyeblink reduction method for the trEEGrid channels, as most regression methods work best with full cap EEG and the trEEGrid does not have the ideal channel density and layout for source separation methods like independent component analysis (ICA [53]).

The significant interaction between electrode and difficulty level for low beta power is difficult to interpret, but may be related to the Fpz_M2 electrode being slightly lower/more frontal and therefore better positioned to record slight differences in low beta power. This is why all conditions differed in the *t*-tests at Fpz_M2 but not at Fpz. Additionally, the Fpz ring electrode was replaced between day 1 and 2, leading to slight variability in the location.

### 4.4. Sleep and Questionnaires

Sleep data were successfully recorded from all subjects, except two subjects due to technical issues, and sleep staged by an expert, similar to previous studies using an early trEEGrid prototype [7,54] or cEEGrid [6]. Even though the sleep expert was trained on traditional electrode configurations used for polysomnography, the expert was capable of extracting hypnograms from the data recorded with the trEEGrid patch. Sleep staging was not affected by single bad channels in the same way as the other tasks, because the process of sleep staging does not tend to rely on a single channel. However, the battery life of the amplifier prototype is not yet long enough to record EEG for the whole night along with impedance, as the battery ran low towards the end of the recording and the EEG stream was cut short in some cases. This technical runtime limitation was known when designing the study. However, the aim of the study was not solely on sleep but on long-term EEG recording during various tasks of daily activity, one of them being sleep. The sleep recording presented here is more of an exploratory nature to highlight the general feasibility of recording sleep EEG with the mobile EEG system presented in this paper. For proper full-length sleep recordings, the battery life should be extended in a next version of the amplifier system. Additionally, this trEEGrid patch version should be validated with a direct comparison with traditional polysomnography as done successfully for a previous version of the patch in da Silva Souto et al. [7].

The answers to the questionnaires indicate that participants slept reasonably well. Additionally, participants report high wearing comfort which did not change over time. Taken together these questionnaire results along with the sleep data demonstrate that the system is comfortable enough to be worn long-term and in overnight EEG recordings. The trEEGrid patch system is much less bulky and cumbersome than traditional polysomnography, which requires electrodes pasted to the participants head and face with sticky paste along with layers of stretchy fabric and tape to keep it all in place. Therefore, this miniaturized system can greatly improve EEG sleep recordings in various use cases and applications in the future.

### 4.5. Limitations

The intention of this study was to explore the feasibility of long-term EEG recording with a new, prototypical mobile EEG recording system during various tasks of daily activity. On the one hand it broadens the horizon regarding different use cases or areas of application such as measuring cognitive functions as well as brain activity while sleeping. On the other hand, this introduces some limitation, as the results presented here lack a certain degree of specificity because several aspects of daily life were addressed. The first step was to show general feasibility; whereas, future studies should specifically address in more detail one of the measures reported here.

Another limitation was that we recorded the EEG tasks in the afternoon on day 1 and in the morning on day 2, in order to have our participants wearing the technology for approximately 24 h. While no observable differences between days were found in the ERP tasks, this time difference appeared to have significantly affected the tasks with spectral activity measures (i.e., resting state and Sudoku), making it difficult to draw conclusions concerning spectral power stability over time, outside of the ability to see task-related effects on both days. We will take this into account for future studies.

The fact that the miniaturized amplifier, the base unit with the battery and the trEEGrid patch used in this study are still in prototype stages leads to some technical limitations. For example, the battery life of the amplifier is limited to 7.5–8 h. Additionally, the connection between the trEEGrid patch system and miniaturized amplifier was somewhat delicate and easy to damage, leading to some data loss. This has already been improved in later iterations of the trEEGrid patch.

While wearing comfort was reported rather favorably in our questionnaires, we must note that all participants were employees or students in the laboratory where this study was performed. Therefore, despite being encouraged to be critical, honest and note any possible issues, these participants may have been biased towards rating the system favorably. Additionally, compared to laypeople, participants were familiar with the comfort or non-comfort of a standard EEG cap to use as reference for comparison. This limits the generalizability of these results, as the assessments of laypeople may differ from those with EEG experience, as presented here.

One final point worth mentioning is that the EEG measurements in this study were conducted under stationary conditions. That is, participants were sitting during the cognitive tasks, and they were lying in bed for the EEG sleep recordings. In dynamic scenarios involving some movement, cables will move and possibly cause greater interference due to artifacts. Artifacts could be more prominent in this system given the higher impedance values compared to wet electrodes prepared with gel.

### 4.6. Future Work

In future work we would like to collect additional data from more naive individuals without prior EEG experience, and also explicitly study the effectiveness of these trEEGrid patch technologies in mobile and dynamic scenarios, as mentioned above. Additionally, we have already started working on new trEEGrid design iterations with multiple frontal channels or an additional channel behind the ear. This way, a certain level of redundancy is achieved and lost connection at one channel will not lead to the loss of an entire dataset for the given analysis. The reduced channel system has drawbacks regarding spatial coverage and as consequence makes the removal of artifacts more difficult; however, this is a trade-off with the comfort and unobtrusiveness of a system that can be worn for many hours without significant discomfort. To address issues with artifact correction, we plan to develop and test different methods of eyeblink and artifact removal to more effectively remove artifact for our trEEGrids and trEEGrid patch data. This includes reduced-channel ICA [53] or regression-based methods [55], which typically require more channel density to be accurate. The combination of increased channel density with newer trEEGrid variations and specialized reduced-channel artifact correction could lead to excellent data processing for future studies. Also, a dedicated study on the quality of the trEEGrid patch for sleep applications should be conducted to enable direct comparisons to standard polysomnography methodologies.

## 5. Conclusions

The trEEGrid patch with miniaturized amplifier system was worn for nearly 24 h and used for brain activity recording in the afternoon, through the night and the next morning. As a response to the first hypothesis, the system was able to demonstrate robust brain signals even with relatively high impedance, including the visual and auditory oddball P3, as well as alpha power changes with eyes closed. With respect to the second hypothesis, we were also able to record EEG data reasonably well during sleep to show sleep stages and during a common cognitive task to show changes in cognitive workload. And finally with respect to the third hypothesis, we received positive feedback (albeit from lab members instead of naïve users) on trEEGrid patch wearing comfort over nearly 24 h as well as sleep quality. The trEEGrid patch system with miniaturized amplifier demonstrated significant signal quality and will be continually improved for future iterations. The results presented here are promising for a new lightweight, mobile EEG recording system delivering multichannel EEG data of high signal quality.

## Figures and Tables

**Table 1:** Overview of wearing time, recording time and analysis inclusion for all participants. For analysis inclusion, filled circles indicate that the participant was included, and non-filled circles indicate that the participant was excluded for the given analysis. The total *n* for each analysis is indicated on the far right.

Total Time (h)	S01	S02	S03	S04	S05	S06	S07	S08	S09	S10	Mean
Wearing	23.67	21.16	20.98	22.80	22.31	23.28	23.53	22.67	22.91	23.49	22.68
Recording	9.67	9.89	8.01	10.10	10.05	9.49	3.55	8.94	10.74	10.26	9.07
**Inclusion in analyses**											**Total**
Impedances	⬤	⬤	⬤	⬤	⬤	⬤	⬤	⬤	◯	⬤	9
Sleep Impedance	⬤	⬤	⬤	◯	⬤	⬤	◯	⬤	◯	⬤	7
Sleep Staging	⬤	⬤	⬤	⬤	⬤	⬤	◯	⬤	◯	⬤	8
SNR	⬤	⬤	⬤	◯	⬤	⬤	⬤	⬤	◯	⬤	8
N1	⬤	⬤	⬤	◯	⬤	⬤	⬤	⬤	◯	⬤	8
Auditory P3	⬤	⬤	⬤	⬤	⬤	⬤	⬤	⬤	◯	⬤	9
Visual P3	⬤	⬤	⬤	⬤	◯	⬤	⬤	⬤	◯	⬤	8
Resting state	⬤	⬤	⬤	◯	⬤	⬤	⬤	⬤	◯	⬤	8
Sudoku	⬤	⬤	⬤	◯	⬤	⬤	⬤	⬤	◯	⬤	8
Questionnaires	⬤	⬤	⬤	⬤	⬤	⬤	⬤	⬤	⬤	⬤	8

**Table 2:** Overview of percentage of impedance values higher than outlier threshold of 200 kOhm for all channels of the trEEGrid and for all participants, S09 excluded. All values higher than ten percent are marked in bold.

	R1	R2	R3	R4	R5	R6	R7	R8
S01	0.54	0.36	0.32	**18.18**	0.23	0.35	**15.72**	**10.27**
S02	2.84	1.35	1.35	0.90	2.17	9.77	1.31	1.04
S03	0.00	0.08	0.01	0.00	0.01	0.00	0.00	0.00
S04	**17.70**	8.41	1.93	**11.36**	0.34	1.66	2.41	0.69
S05	0.00	0.00	0.00	0.00	0.00	0.00	0.00	0.00
S06	0.00	0.00	0.00	0.00	0.00	0.00	0.00	0.00
S07	0.00	0.05	0.00	0.05	**99.73**	0.05	0.00	0.00
S08	1.27	0.00	0.00	0.00	**49.30**	**12.06**	0.00	0.00
S10	3.61	3.50	6.77	**19.60**	3.51	4.33	4.80	6.22

## Data Availability

The raw data supporting the conclusions of this article will be made available by the authors, upon request and in accordance with the consent of the participants as per the data protection agreement.

## Supplementary Materials

The following supporting information can be downloaded at https://www.mdpi.com/article/10.3390/s26165309/s1.

## Abbreviations

The following abbreviations are used in this manuscript:

AASM	American Academy of Sleep Medicine
ANOVA	Analysis of Variance
ASR	Artifact-Subspace-Reconstruction
EDF	European Data Format
EEG	Electroencephalogram
EOG	Electrooculogram
ICA	Independent component analysis
