# Supplementary material for: Around the Clock: Wearing a Flex-Printed trEEGrid Electrode Patch and Miniaturized Amplifier for Nearly 24 Hours Across Daytime and Overnight EEG Sessions
Source: Sensors (Basel). 2026 Aug 21;26(16):5309. doi: 10.3390/s26165309 (PMC13517458; doi:10.3390/s26165309)
Supplement: Supplementary file 1 [file sensors-26-05309-s001.zip › sensors-4429917-supplementary.pdf]

Supplementary Materials

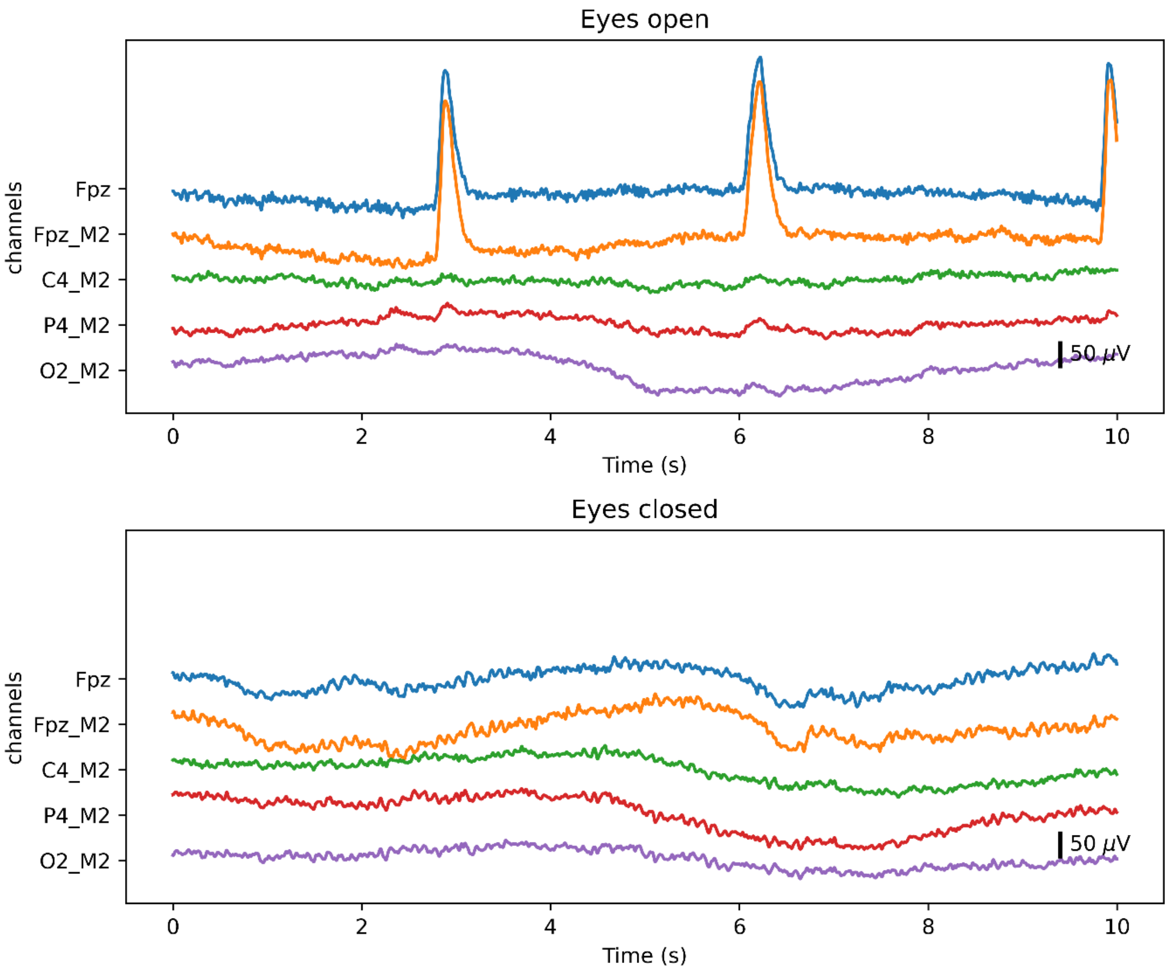

Supplementary Figure S1. Raw data from the resting state task, participant S05, day 1.

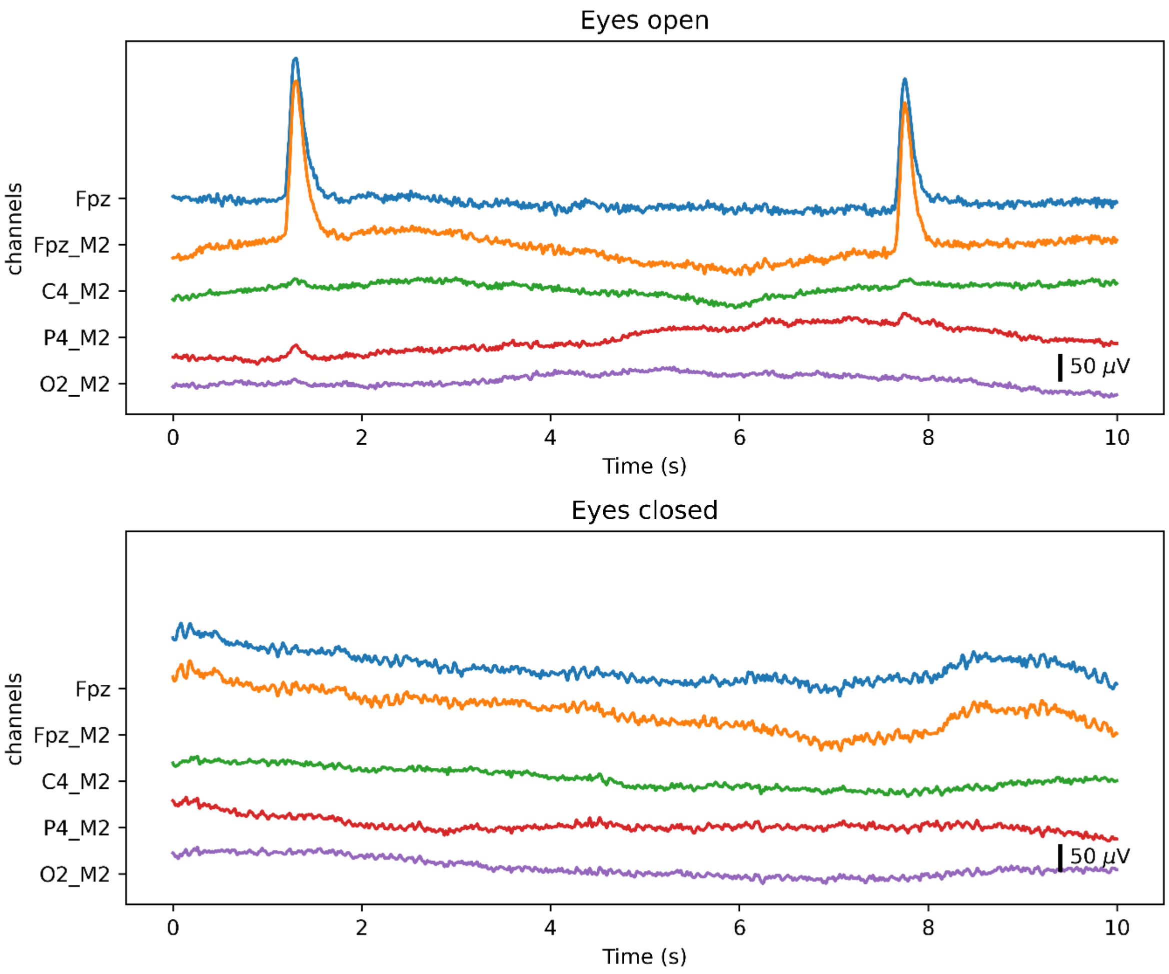

Supplementary Figure S2. Raw data from the resting state task, participant S05, day 2.

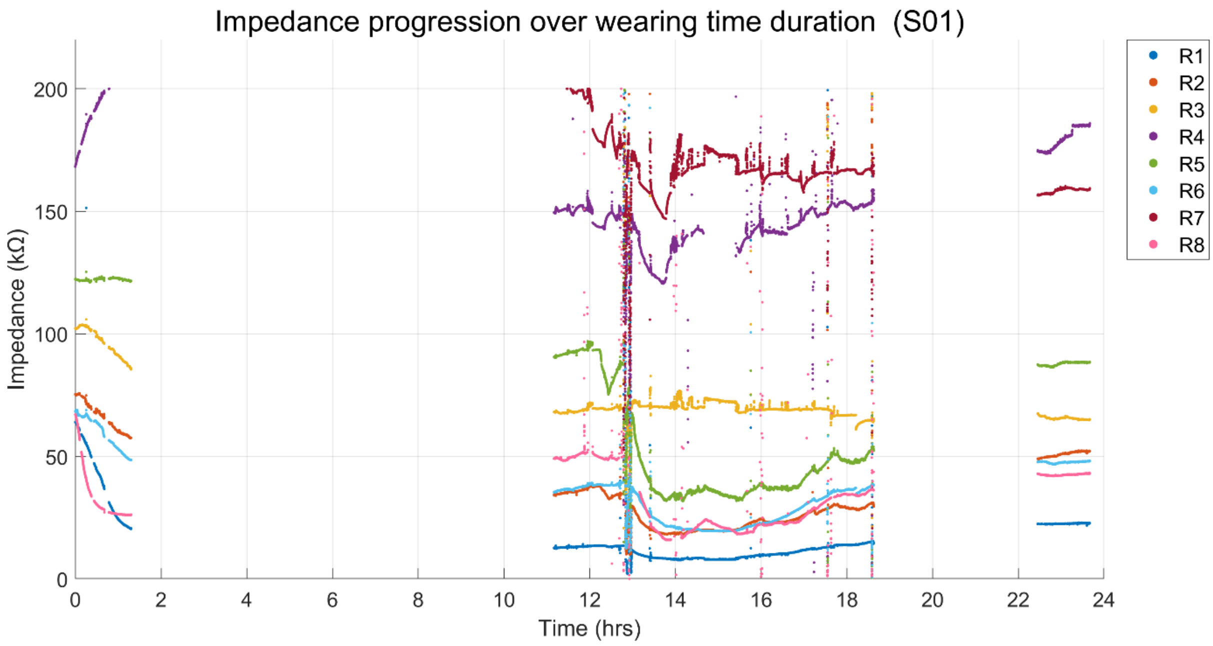

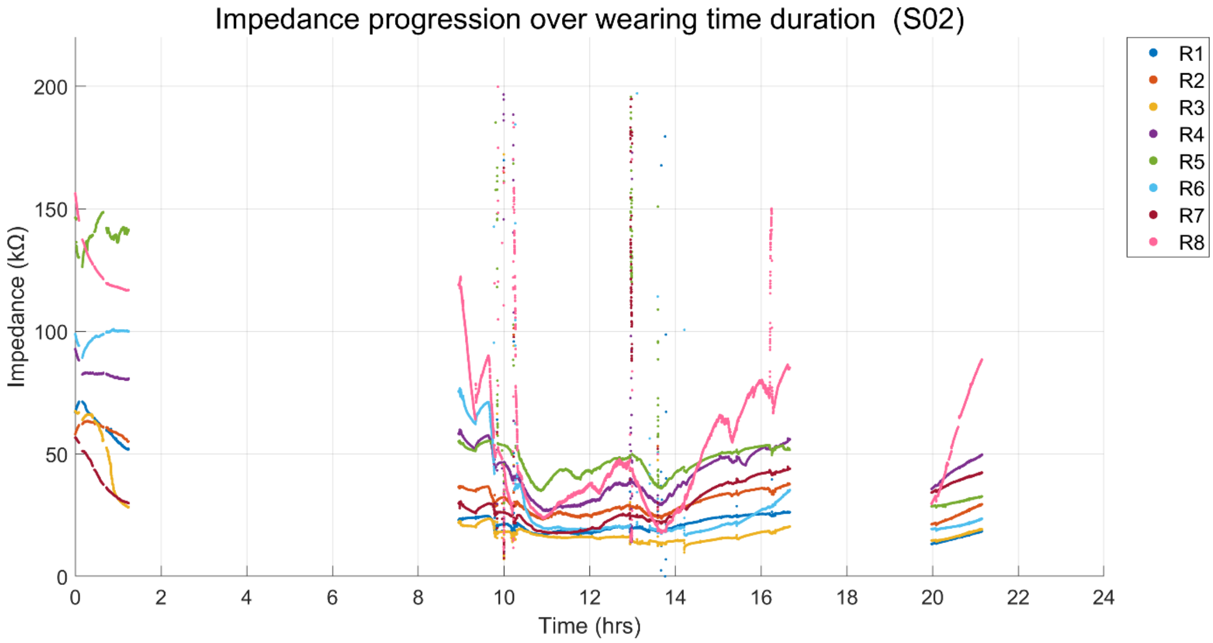
Impedance progression over wearing time duration (S02)
Impedance (kΩ)
Time (hrs)
0
50
100
150
200
0
2
4
6
8
10
12
14
16
18
20
22
24
R1
R2
R3
R4
R5
R6
R7
R8

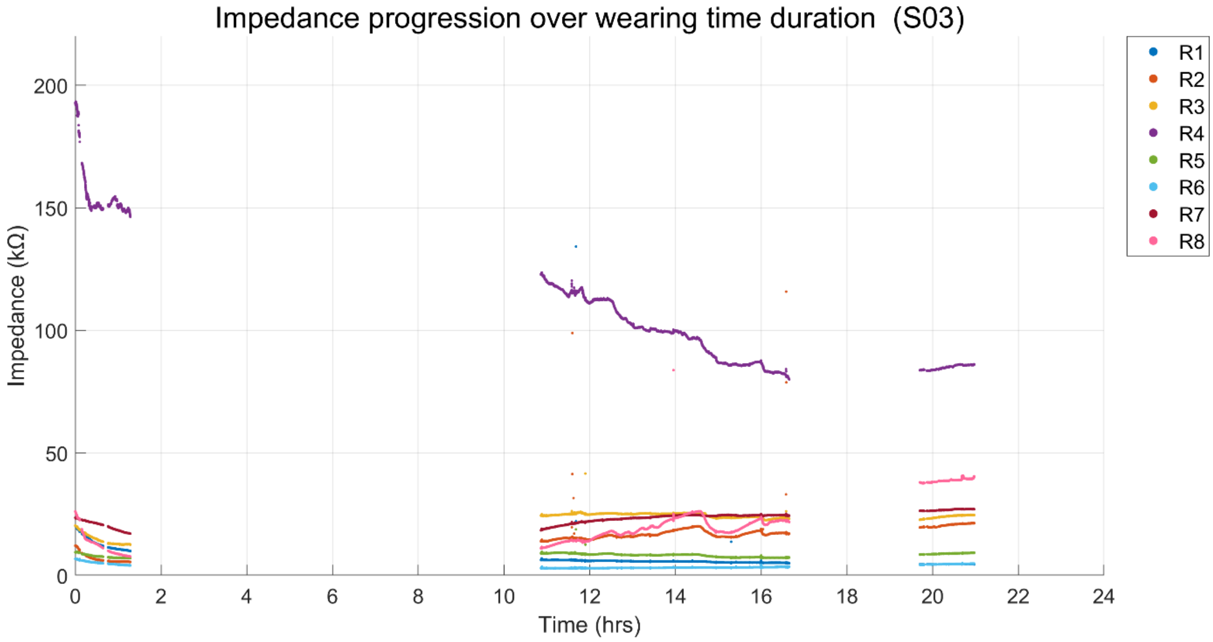
Impedance progression over wearing time duration (S03)
Impedance (kΩ)
Time (hrs)
0
50
100
150
200
0
2
4
6
8
10
12
14
16
18
20
22
24
R1
R2
R3
R4
R5
R6
R7
R8

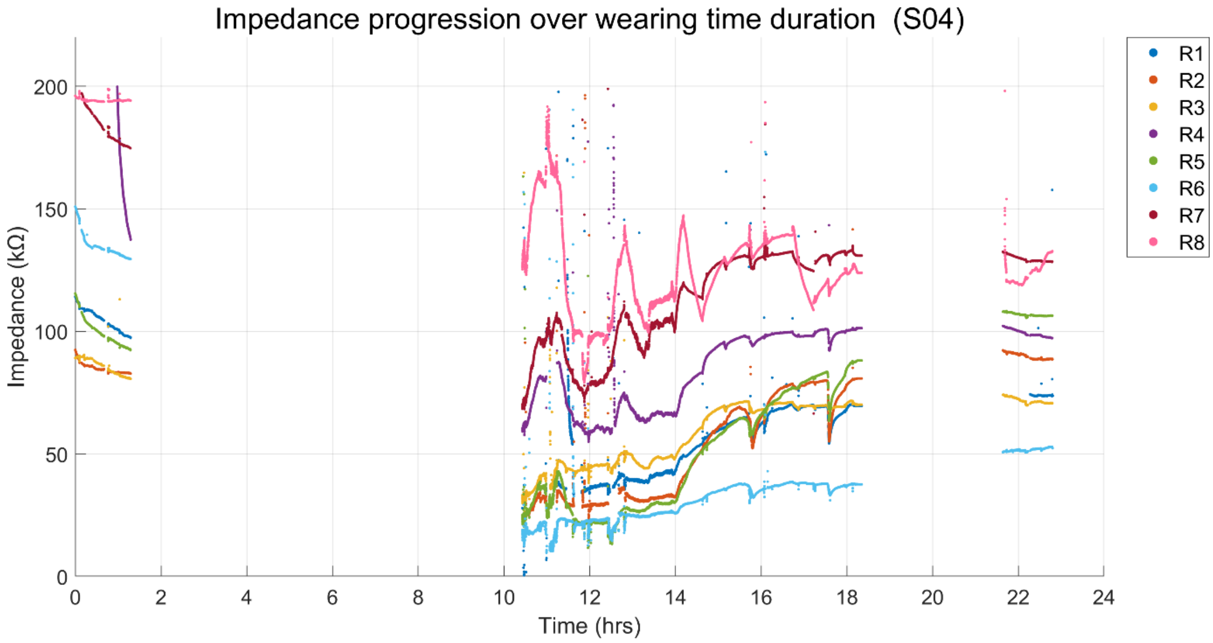

Impedance progression over wearing time duration (S04)
Impedance (kΩ)
Time (hrs)
0
50
100
150
200
0
2
4
6
8
10
12
14
16
18
20
22
24
R1
R2
R3
R4
R5
R6
R7
R8

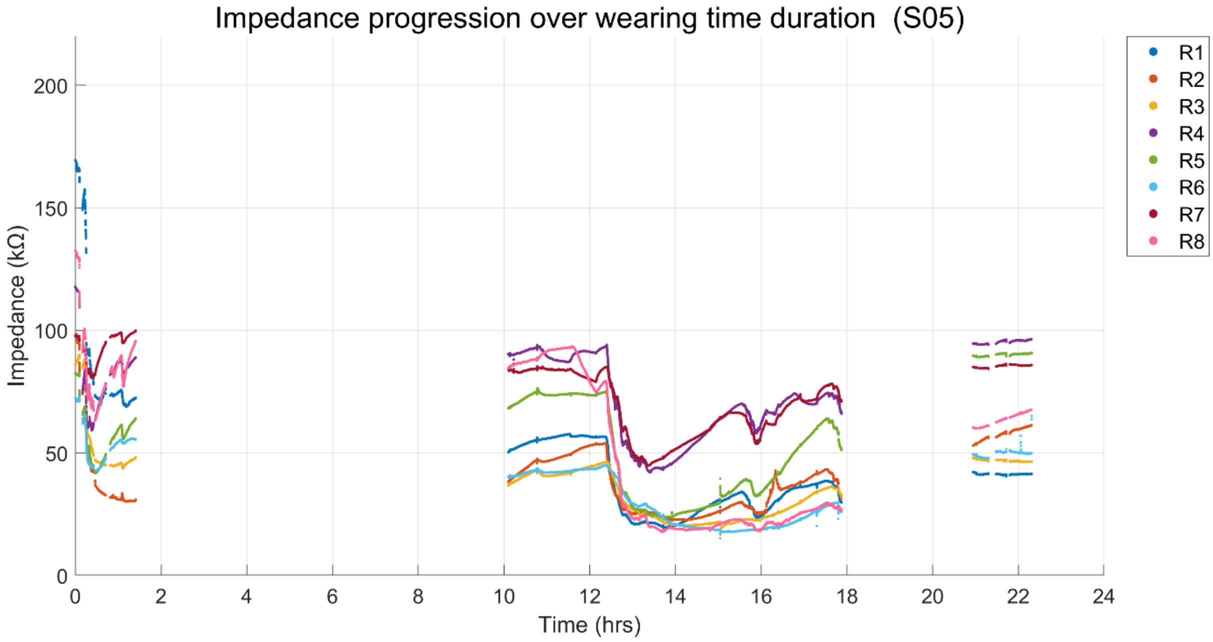

Impedance progression over wearing time duration (S05)
Impedance (kΩ)
Time (hrs)
0
50
100
150
200
0
2
4
6
8
10
12
14
16
18
20
22
24
R1
R2
R3
R4
R5
R6
R7
R8

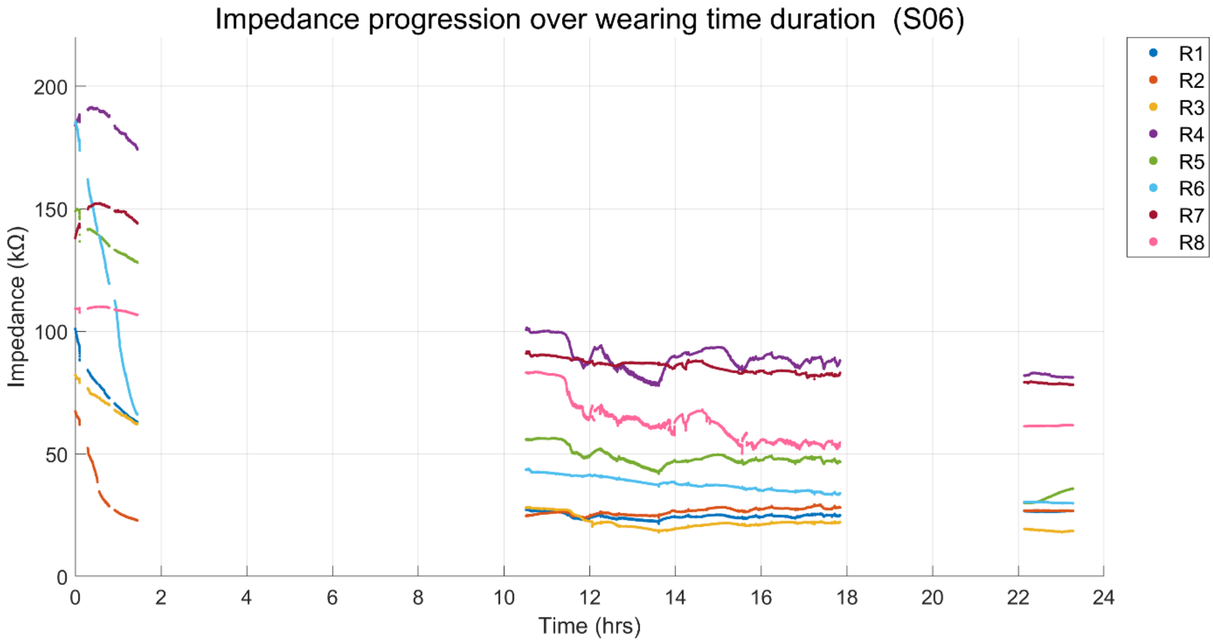
Impedance progression over wearing time duration (S06)
Impedance (kΩ)
Time (hrs)
R1
R2
R3
R4
R5
R6
R7
R8
0
50
100
150
200
0
2
4
6
8
10
12
14
16
18
20
22
24

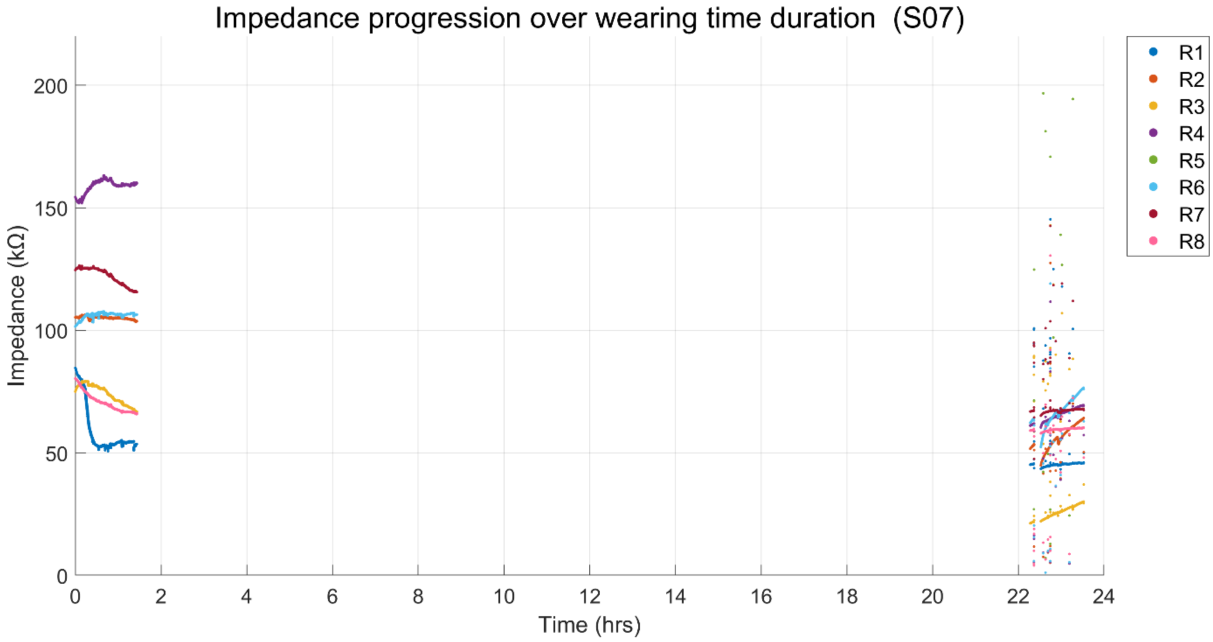
Impedance progression over wearing time duration (S07)
Impedance (kΩ)
Time (hrs)
R1
R2
R3
R4
R5
R6
R7
R8
0
50
100
150
200
0
2
4
6
8
10
12
14
16
18
20
22
24

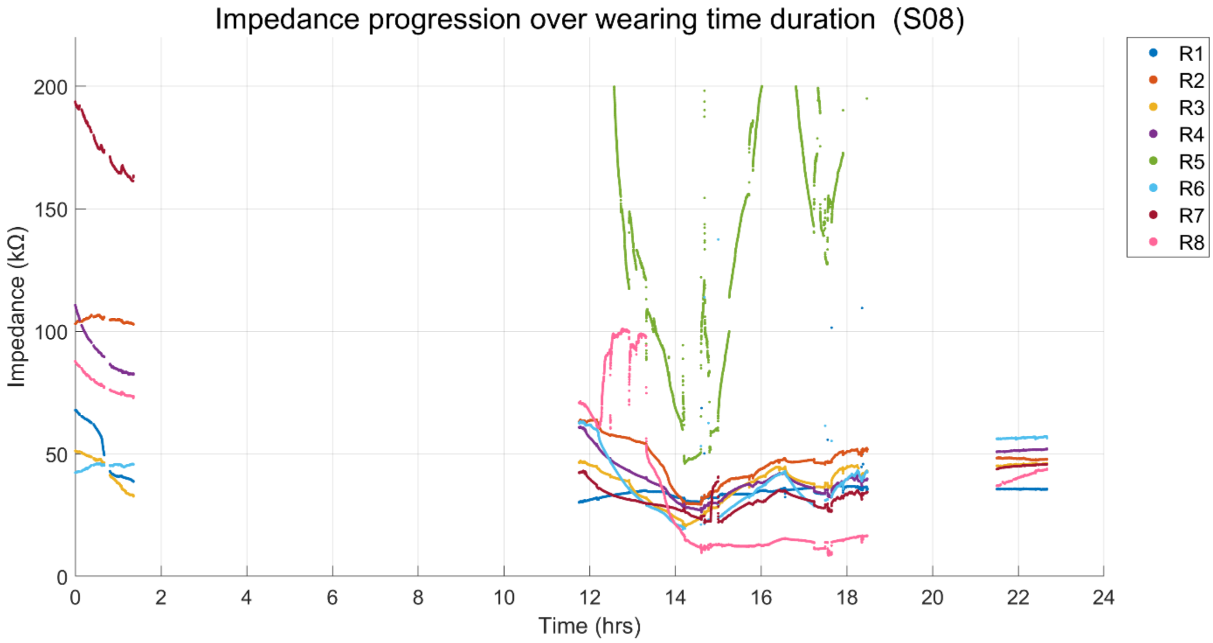

Impedance progression over wearing time duration (S08)
Impedance (kΩ)
Time (hrs)
R1
R2
R3
R4
R5
R6
R7
R8

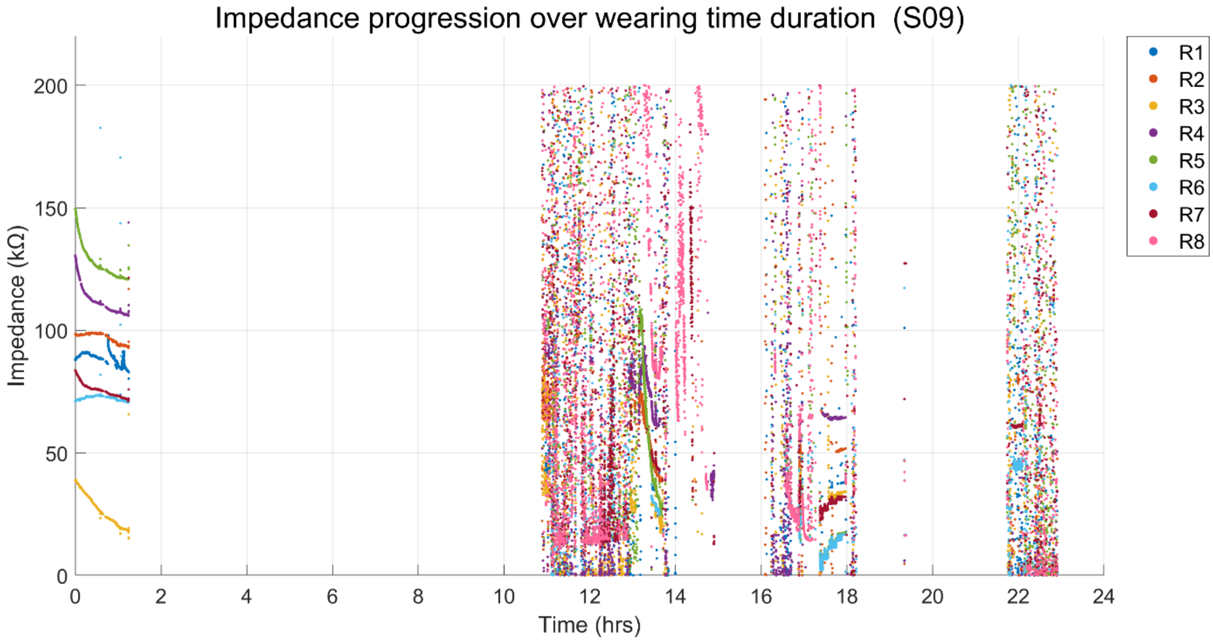

Impedance progression over wearing time duration (S09)
Impedance (kΩ)
Time (hrs)
R1
R2
R3
R4
R5
R6
R7
R8

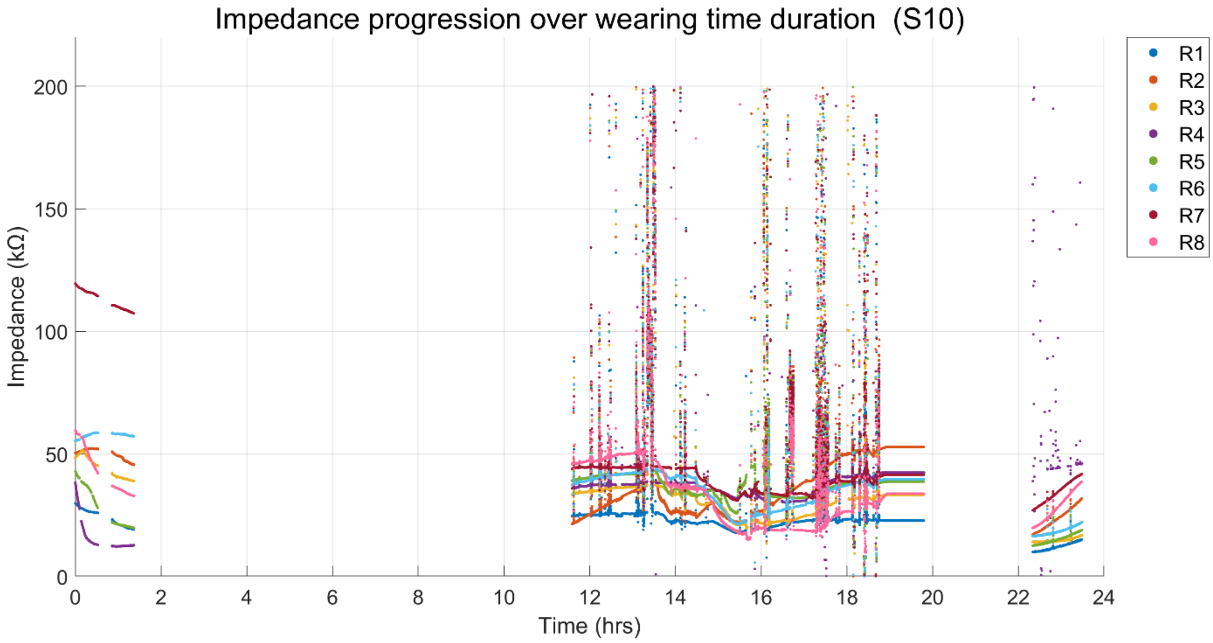

Supplementary Figure S3. Impedance progression for each participant over the course of a nearly 24-hour recording and wearing the trEEGrid patch. For subject S07 the night recording is missing due to technical recording problems. The recording of subject S09 is probably affected by a damaged connector on the trEEGrid patch for the connection to the amplifier.
